# Resolving and correcting for kinetic biases on methane seep paleotemperature using carbonate ∆_47_/∆_48_ analysis

**DOI:** 10.1126/sciadv.adn0155

**Published:** 2024-05-29

**Authors:** Philip Staudigel, Dong Feng, Jörn Peckmann, Miguel Bernecker, Amelia Davies, Mattia Tagliavento, Jens Fiebig

**Affiliations:** ^1^ Goethe-Universität Frankfurt, Institut für Geowissenschaften, Frankfurt am Main 60438, Germany.; ^2^ Shanghai Ocean University, College of Marine Sciences, Shanghai 201306, China.; ^3^ Universität Hamburg, Institute for Geology, Center for Earth System Research and Sustainability, Hamburg 20146, Germany.; ^4^ Universität zu Köln, Institut für Geologie und Mineralogie, Köln 50939, Germany.; ^5^ Division of Geological and Planetary Sciences, Caltech, Pasadena, CA 91125, USA.

## Abstract

Methane-derived authigenic carbonate often constitutes the sole remaining record of relic methane seeps. The clumped (∆_47_) and oxygen isotopic composition of seep carbonates often yield inaccurate temperatures, attributed to kinetic isotope effects and modification of seawater isotope composition by hydrate water. Here, we analyzed the dual-clumped isotope (∆_47_/∆_48_) composition of authigenic carbonate from a modern methane seep. We demonstrate that aragonite forms closest to isotopic equilibrium such that its ∆_47_ can directly yield the correct formational temperature, whereas calcite is unambiguously biased by kinetic isotope effects. Numerical models show that the observed bias in the isotopic composition arises from rate-limiting dehydration/dehydroxylation of HCO_3_^−^ alongside diffusive fractionation, which can be corrected for with analysis of carbonate ∆_47_/∆_48_ values. We demonstrate the utility of dual-clumped isotope analysis for studying seep carbonates, as it reveals the origin and magnitude of kinetic biases and can be used to reconstruct paleotemperature and seawater δ^18^O.

## Teaser

Kinetic biases on methane seep carbonate paleotemperatures can be quantified and corrected for using dual-clumped isotopes.

## INTRODUCTION

The global inventory of methane hydrate has been estimated through extrapolation of field observations (*1*, *2*), as well as numerical climate modeling (*3*–*5*). These estimates range between 1000 and 20,000 GtC within 2 km of the Earth’s surface, establishing methane hydrates as a substantial reservoir of reduced carbon, similar in size to that of global petroleum deposits and up to half the total dissolved inorganic carbon (DIC) in the ocean-atmosphere system. This methane is, for the moment, sequestered in terrestrial and marine sediments (*3*, *6*). The release of this methane is not always a continuous process, for example, slope failures on continental margins can result in substantial releases of methane over relatively short timescales (*7*). Long-term changes to this inventory are likewise an important variable in climate models, as even minor changes [e.g., 0.05% over 100 years (*8*)] would constitute an additional flux of carbon of ~0.05 GtC/year, roughly equivalent in magnitude to modern volcanism (*9*), but considerably less than modern fossil fuel use [estimated 10.0 GtC released in 2022 (*10*)]. Such a flux is nevertheless important, as it would persist thousands of years after direct fossil fuel use is eliminated, making it an important variable when describing humanity’s long-term contributions to the Earth’s carbon budget and climate.

Methane hydrate stability increases with pressure and decreases with temperature (*6*, *11*); thus, changes in the magnitude of methane degassing reflect changes in local and global conditions. The destabilization of methane hydrates is understood as a positive feedback mechanism during environmental warming in the past and present (*12*, *13*). Warming of intermediate water masses during recent deglacial intervals can be associated with periods of methane seepage in the South China Sea (*14*) and Gulf of Guinea (*15*), and increased methane seep activity at high latitudes is likewise linked to deglaciations (*16*). The precise mechanisms driving this linkage are not always obvious, particularly in regions where isostatic uplift during deglaciation can outpace sea-level rise, resulting in a drop in confining pressure in addition to the water mass warming (*17*). This sensitivity to both global and local factors makes methane seeps a challenging variable in climate models (*8*, *13*). Accurately reconstructing the physical and chemical conditions in relic seep systems is key to understanding how seeps responded to perturbations in temperature and pressure in the past, providing valuable context for predicting their long-term response to present climate change.

**Fig. 1.**
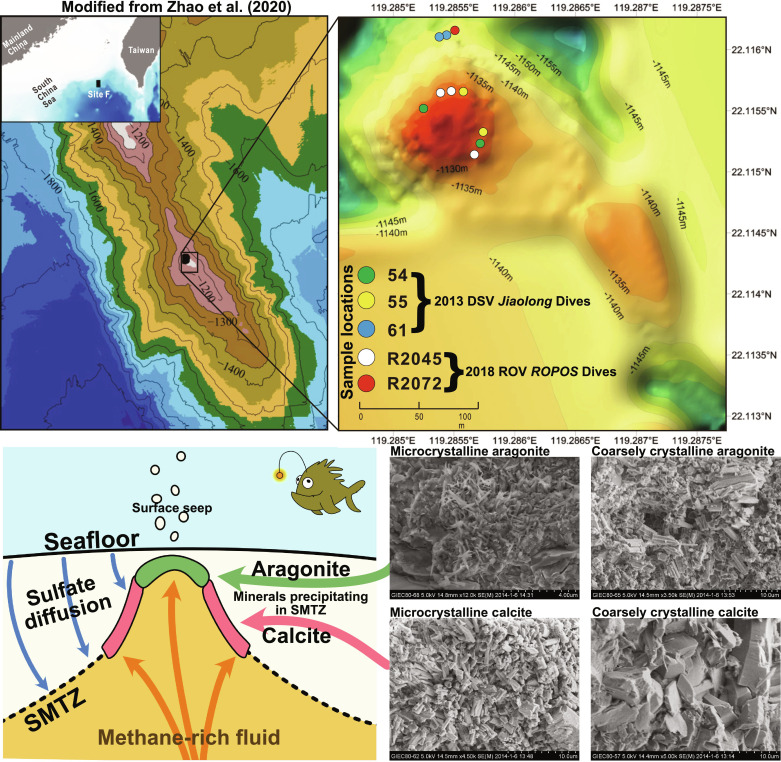
Sample site location, background information, and petrography. (**Top**) Map, modified from Zhao *et al.* (*52*), showing location where samples were collected with separate dive sample locations shown. (**Bottom left**) Schematic diagram showing the sulfate-methane transition zone (SMTZ) and the zones of aragonite and calcite formation. (**Bottom right**) SEM images of the different materials sampled in this study, microcrystalline, and coarsely crystalline calcite and aragonite.

Carbonate minerals are an abundant by-product of sulfate-driven anaerobic oxidation of methane (SD-AOM) (*18*), and mineralization rates are greatest in regions where sulfate and methane react [sulfate-methane transition zone (SMTZ); Fig. 1]. These minerals constitute a geochemical archive of ancient seep conditions, as their isotopic properties reflect the origin of carbon (reflected in carbonate δ^13^C values), as well as water properties and temperature (recorded in δ^18^O and ∆_47_ values). Oxygen isotope variability in methane-derived carbonates has frequently been attributed to the enrichment of ^18^O in methane hydrates (*19*–*21*); however, analyses of clumped isotopes reveal prominent correlated changes in δ^18^O and ∆_47_ values, suggesting that a kinetically controlled process may have affected these values, with sufficient disequilibrium to account for the observed ~1‰ enrichment in δ^18^O values (*22*–*24*). This kinetic hypothesis presents a challenge for researchers seeking to reconstruct relic seep conditions, as the magnitude of this kinetic isotope effect (KIE) is not constant between samples, and thus makes reconstructing seep temperature using conventional clumped isotopes impossible without some a priori knowledge of water δ^18^O values or some other constraint.

The rate-limiting chemical reactions for equilibrating DIC are typically the (de)hydration and (de)hydroxylation reactions between HCO_3_^−^ and CO_2_ (*25*), which both involve the formation or breaking of a covalent bond and are thus slower than the protonation reactions, which govern bicarbonate/carbonate equilibrium. In systems that are chemically perturbed (e.g., DIC being added or removed), KIEs associated with these reactions can be observed (*26*, *27*). Mass-dependent fractionation during diffusion further affects the isotopic composition of DIC, arising from the more rapid diffusion of lighter isotopologues of DIC species (*22*, *28*). This disequilibrium state of DIC is inherited by precipitating carbonate minerals. If minerals precipitate rapidly, additional fractionation can occur due to KIEs at the mineral-fluid boundary (*26*). Many factors can simultaneously affect the isotopic composition of DIC and precipitating minerals; thus, methane seeps can differ compositionally from region to region, presumably reflecting different contributions of these effects (*22*, *23*, *29*).

Because of the variable expression of KIEs, calculating paleotemperatures and interpreting the physical-chemical state of relic seep systems would yield ambiguous results dependent upon many assumptions regarding the magnitude of these offsets. Recent advances in clumped isotopes (*30*) have shown that with the analyses of multiple isotopologues at mass/charge ratio (*m*/*z*) = 47 (∆_47_) and *m*/*z* = 48 (∆_48_), the effects of these kinetic processes can be resolved (*31*–*33*), allowing for more confident reconstruction of temperatures (*34*–*36*), as well as identifying diagenetic effects (*33*, *37*, *38*), and fingerprinting the processes that caused the kinetic bias (*26*, *27*, *32*, *35*, *38*, *39*).

To test the feasibility of using coupled ∆_47_/∆_48_ analyses for identifying kinetic biases in methane seep carbonates, we have analyzed 10 carbonate-rich samples from a modern methane seep (site F, also known as Formosa Ridge, South China Sea; Fig. 1). The recently published numerical framework from Watkins and Devriendt (*26*) [carbon oxygen α Δ: (COAD) model] allows for the simulation of KIEs in both the dissolved carbonate phase and the kinetic effects during mineral formation, yielding predictions for the kinetic behavior of conventional and dual-clumped isotopologues. We have modified the COAD to create the COAD Methane Seep (COAD MS) model, which simulates dissolved methane, sulfate, sulfide, molecular oxygen, and alkalinity, which has produced an implementation that allows solution chemistry, including the pH, to vary. The processes at seeps are simulated as a zero-dimensional reactive box model, wherein seawater and methane are mixed at a user-defined mixing rate (λ) and allowed to react until steady state is achieved. With slower mixing rates, methane is given sufficient time to react with oxygen, and then with sulfate, resulting in the production of DIC, which then equilibrates following the predictions of the COAD model and reacts with dissolved Ca^2+^ to form carbonate minerals. The modeled offsets in δ^18^O, ∆_47_, and ∆_48_ values from equilibrium are compared to our measured carbonate samples from site F.

## RESULTS

**Fig. 2.**
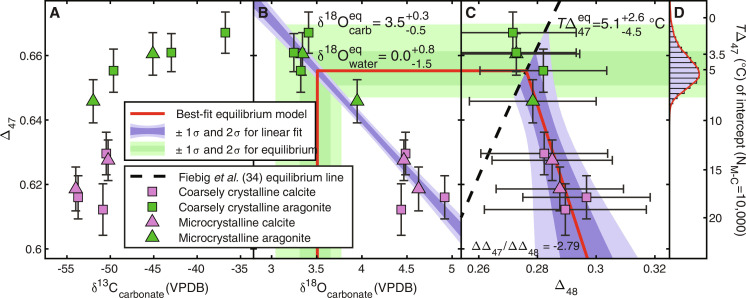
Measured isotope data from methane seep carbonates. Measured data plotted as symbols, with error bars showing ±95% confidence of measurement. Blue shaded regions show 1σ and 2σ confidence for linear regressions. Green shaded regions show 1σ and 2σ confidence for equilibrium temperature and carbonate δ^18^O composition based on intercept with ∆_47_/∆_48_ equilibrium line. Uncertainties for regressions and equilibrium state were calculated via 10,000 Monte-Carlo iterations for linear regression accounting for measurement uncertainties in ∆_47_ and ∆_48_. (**A**) δ^13^C and ∆_47_ values. (**B**) δ^18^O and ∆_47_ values. Shaded regions show 1σ and 2σ confidence for equilibrium temperature and δ^18^O values and covariance between ∆_47_ and δ^18^O. (**C**) ∆_48_ and ∆_47_ values. Red line in (B) and (C) graphically represents the conversion from slope of the ∆_47_/∆_48_ data to an equilibrium intercept, which is then used to calculate the equilibrium δ^18^O value for carbonate and water. Green shaded region shows 1σ and 2σ confidence for intercept between linear fit and Fiebig *et al.* (*34*) equilibrium ∆_47_/∆_48_ relationship. (**D**) Histogram and kernel density function showing probability of intercepts of ∆_47_/∆_48_ linear regression with the equilibrium temperature line as calculated using 10,000 iterations of a Monte-Carlo simulation.

**Fig. 3.**
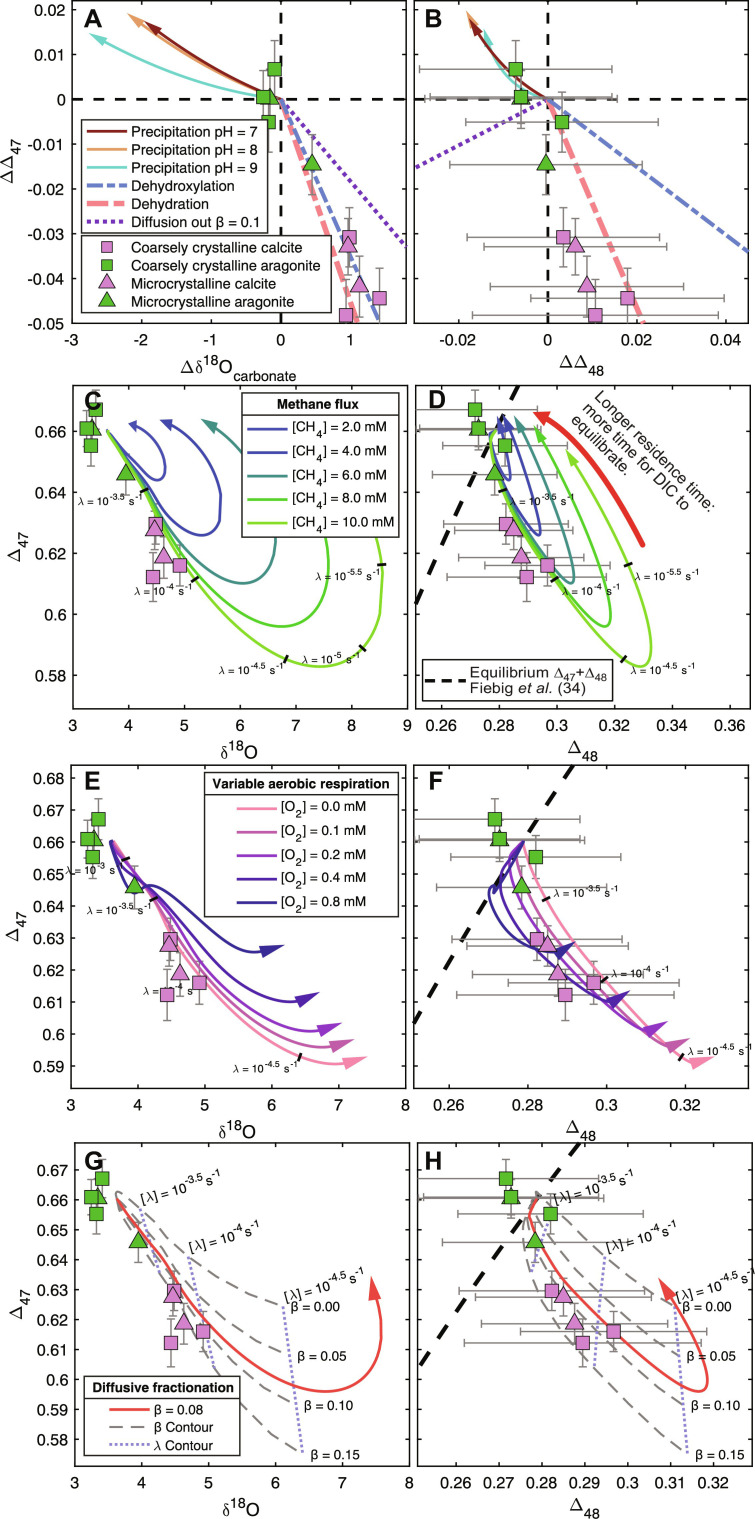
Model output and isotopic results. (**A** and **B**) Effect of rapid carbonate precipitation calculated using the model of Watkins and Devriendt (*26*), dehydration, dehydroxylation, and diffusion on carbonate δ^18^O, ∆_47_, and ∆_48_ values. Carbonate precipitation vectors show precipitation from equilibrium to 10^−4^ mol m^−2^ s^−1^. Data shown relative to the extrapolated “equilibrium” composition calculated in Fig. 2. Models showing the effect of decreasing λ given different model settings are shown as arrows. (**C** and **D**) Variable methane concentrations’ effect on output values. (**E** and **F**) Variable dissolved O_2_ concentrations’ effect on output values. (**G** and **H**) Varying the intensity mass-dependent fractionation during DIC diffusion (β value). Measured data plotted as symbols, with error bars showing ±95% confidence.

The samples from Formosa Ridge contained between 70 and 100 wt % aragonite or calcite. Carbonate isotope results are shown in Fig. 2 and Table 1. Carbonate δ^13^C values were between −35 and −55‰ relative to the Vienna Pee Dee Belemnite isotope standard (VPDB) (Fig. 2A), with δ^18^O values ranging between 3 and 5‰ VPDB (Fig. 2B). Clumped isotope composition ranged from 0.61‰ to 0.67‰ and 0.27‰ to 0.30‰ for ∆_47_ and ∆_48_, respectively, corresponding to a temperature range of 2° to 19°C and −5° to 8°C, respectively. All aragonite samples were indistinguishable from ∆_47_/∆_48_ equilibrium and yielded an average *T*(∆_47_) of 4.4°C, and most calcite samples were distinct from ∆_47_/∆_48_ equilibrium with an average *T*∆_47_ of 16°C (Fig. 2C). There is no significant difference between microcrystalline and macrocrystalline samples (*P* > 0.05, Welch’s *t* test) squares and triangles in Figs. 2 and 3), although the difference between calcite and aragonite samples remains statistically significant (*P* < 0.01, Welch’s *t* test).

**Table 1.** Carbonate isotope analysis results. Bleached samples are duplicates. ±95% CL values represent fully error propagated 2 SE.

Sample type	Dive ID	Water depth (m)	*N*	δ^13^C ‰ VPDB	δ^18^O ‰ VPDB*	∆_47_ (‰ CDES-90)	±95% CL	∆_48_ (‰ CDES-90)	±95% CL
*Jiaolong* 2013 Dives (microcrystalline)									
Calcite	55-3	1146	8	−53.71	4.91	0.616	0.007	0.297	0.022
Aragonite	55-2	1126	8	−49.63	3.32	0.655	0.007	0.282	0.022
Aragonite	54-1	1126	8	−36.75	3.40	0.667	0.006	0.272	0.022
Calcite	61-3	1146	8	−50.47	4.48	0.630	0.007	0.282	0.022
Aragonite	54-2	1126	10	−42.97	3.24	0.661	0.006	0.273	0.020
Calcite	61-2	1146	5	−50.85	4.43	0.612	0.008	0.290	0.028
*ROPOS* 2018 Dives (coarsely crystalline)									
Calcite	R2045-2	1126	8	−53.98	4.62	0.619	0.007	0.288	0.022
Aragonite	R2045-4	1126	8	−45.10	3.33	0.661	0.007	0.273	0.022
Calcite	R2072-3	1146	9	−50.25	4.46	0.628	0.006	0.285	0.021
Aragonite	R2045-3	1126	8	−51.97	3.94	0.646	0.007	0.278	0.022
Bleached samples									
Aragonite-bleached	54-2	1126	8	−42.89	3.18	0.661	0.007	0.287	0.022
Calcite-bleached	61-2	1146	5	−50.66	4.39	0.609	0.008	0.317	0.028
Contamination test									
Carrara Marble			97	1.97	−1.59	0.309	0.002	0.149	0.006
Carrara Marble + 2% pyrite			9	1.92	−1.56	0.309	0.006	0.143	0.021

*δ18O values corrected for acid fractionation at 90°C using the aragonite and calcite fractionation factors of Kim *et al*. (*56*).

Figure 3 (A and B) shows the effects of individual processes that promote KIEs plotted with measured ∆δ^18^O ∆∆_47_ and ∆∆_48,_ normalized to the measured benthic temperature of 3.5°C. Preferential removal of lighter isotopologues of bicarbonate during diffusion results in an enrichment of heavier isotopologues in the residual DIC. The kinetic slope of this effect is shown for δ^18^O, ∆_47_, and ∆_48_. Kinetic fractionation during dehydration and dehydroxylation also yield distinguishable kinetic slopes that are shown in Fig. 3 (A and B). The rate of carbonate formation is simulated using the CaCO3_DIC.m function published by Watkins and Devriendt (*26*). The arrows shown in Fig. 3 (A and B) show the composition of carbonate precipitating from an otherwise equilibrated DIC pool between 0 and 10^−4^ mol m^−2^ s^−1^. These mineral precipitation kinetic models predict positive ∆∆_47_ and negative ∆δ^18^O and ∆∆_48_ offsets for a wide range of pH values.

Models shown in Fig. 3 (C to H) show the steady-state isotopic composition of carbonate for different exchange rates with seawater and the methane-rich fluid (λ). Each of these lines originates at ∆_47_/∆_48_ equilibrium with a λ value of 100 s^−1^, which, when decreased, increases the residence time of reactants, allowing aerobic oxidation of sulfide (AeOS) and SD-AOM to occur. In all models, decreasing the λ value from 10^−3^ s^−1^ to 10^−5^ s^−1^, thus increasing residence time of products and reactants, results in progressively more disequilibrated carbonate δ^18^O, ∆_47_, and ∆_48_ values. In models with the λ values less than 10^−5^ s^−1^ (e.g., those shown in Fig. 3, C and D), the steady-state isotopic composition of the system again approaches isotopic equilibrium, as the physical supply of reactants becomes the rate-limiting process rather than chemical reactions.

Models with greater methane flux exhibited increased disequilibrium of the δ^18^O-∆_47_–∆_48_ system, and models initially followed a similar trend away from equilibrium. Increased oxygen concentrations resulted in a departure from equilibrium moving initially along the equilibrium ∆_47_/∆_48_ line (Fig. 3F). Changes to the diffusive mass-dependent isotopologue fractionation coefficient (β) results in large changes in overall model behavior. Models where β = 0.00 exhibited a nearly linear departure from equilibrium along a slope of −1.75 as λ decreases to 10^−5^ s^−1^, with no AeOM (aerobic oxidation of methane)–associated offsets along the equilibrium line; however, increasing the β value to 0.15 resulted in a larger initial offset (gray dashed lines, Fig. 3, G and H).

## DISCUSSION

### Empirical reconstruction of seep temperature and seawater δ^18^O

The average paleotemperature reconstructed from aragonite ∆_47_ values at Formosa Ridge is 4.4 ± 1.1°C (±1 SE), and all aragonite *T*∆_47_ values except one are indistinguishable from the measured temperature of 3.5°C (Fig. 3, A and B). This observation is further supported by the agreement between ∆_47_ and ∆_48_ temperatures in these samples, which are all indistinguishable from the equilibrium ∆_47_/∆_48_ curve (*34*). Using an aragonite-water specific isotope fractionation equation (*40*), we calculate a δ^18^O_water_ of +0.1 ± 0.4‰ (±1 SE), a value indistinguishable from the local value of −0.3‰ (*20*). Thus, we show that by selecting samples with ∆_47_/∆_48_ agreement, it is possible to reconstruct seep temperatures accurately. Analyses of the calcite components at Formosa Ridge yielded an average ∆_47_ temperature of 16.3 ± 1.1°C (±1 SE), with invariably disequilibrium ∆_47_ and ∆_48_ values, and thus, their corresponding temperatures can be confidently disregarded.

Presuming all samples to have some expression of a kinetic bias, it is possible to construct a linear fit for ∆_47_ and ∆_48_ values, the intercept of which would then extrapolate to the equilibrium temperature (Fig. 2C). For all samples, the slope for ∆_47_/∆_48_ is −2.79 and yields an intercept with the ∆_47_/∆_48_ equilibrium line (*34*) at 5.1°C (red line, Fig. 2C), with a 95% confidence between 0.6°C and +7.7°C (Fig. 2D). This equilibrium endmember has a δ^18^O value of $3.5_{–0.5}^{+0.3}$‰ VPDB, yielding a water isotope ratio of $0.0_{–1.5}^{+0.8}$‰ relative to the Vienna Standard Mean Ocean Water isotope standard (VSMOW) using the Coplen δ^18^O-temperature relationship (*41*). This number can be compared favorably to the local water value of −0.3‰ VSMOW (*20*). This process is shown graphically in Fig. 2 as a red line, where statistics are calculated by repeating this analysis using Monte-Carlo approach, resampling carbonate ∆_47_ and ∆_48_ values following their measured uncertainties.

Previously published studies have found that authigenic aragonite tends to form in the less reducing and more open conditions closer to the seafloor than calcite (Fig. 1) (*20*, *42*, *43*), which at site F appears to be correlated with more isotopically equilibrated compositions. However, mineralogy should not be treated as an unambiguous indicator for equilibrium precipitation, as seep carbonate in the North Sea and Barents Sea have shown the opposite trend, with aragonite yielding invariably disequilibrated temperature (*24*). We therefore propose ∆_47_/∆_48_ analysis as a means of unambiguously identifying kinetic biases in relic seeps, especially when fluid δ^18^O values and precipitation temperatures are unknown.

### Different fluid sources for aragonite and calcite?

The observed dissimilarity in isotopic composition of calcite and aragonite cements in seep systems is commonly observed, which was previously attributed to variable contribution of ^18^O-enriched water from methane hydrates (*21*). The effects of nonlinear mixing of ∆_47_ and ∆_48_ between a δ^13^C-depleted and δ^18^O-enriched masses are evaluated in the Supplementary Materials and could explain the observed offset, but this would require fluids enriched by 10‰ in δ^18^O, which exceeds the measured 3‰ enrichment between hydrates and water. Clumped isotope analyses of seep carbonates show that these δ^18^O offsets are coupled with ∆_47_ offsets (*23*, *29*), although this could possibly be attributed to coupled differences in temperature and fluid composition. Because samples that exhibit δ^18^O offsets simultaneously exhibit ∆_47_/∆_48_ offsets, we argue that this is more likely the result of KIEs present in the seep and does not require variable fluid δ^18^O values.

### Factors contributing to disequilibrium

Samples exhibiting disequilibrium compositions have scientific value beyond simply reconstructing paleotemperatures, as the direction and magnitude of their isotopic disequilibrium can be used to identify physical/chemical processes biasing isotopic composition and thus give insight into the geomicrobiology of the seep environment (Fig. 3, C to H).

Multiple factors can force methane seep carbonates toward isotopic disequilibrium. Several of these were tested using the COAD-MS model. KIEs governing the transport of DIC to the mineral surface and during mineral formation are expected to affect the isotopic composition of carbonate. This is driven by KIEs present at the mineral-water boundary, which are described using the CaCO3_DIC.m function from Watkins and Devriendt (*26*). This process results in positive ∆∆_47_ and negative ∆∆_48_ values with considerable negative offset in ∆δ^18^O, the magnitude of these offsets increases with more rapid precipitation (shown as arrows in Fig. 3, A and B), and the precise direction of this offset varies with solution pH (different colored arrows in Fig. 3, A and B). Nevertheless, the directions and magnitude of these mineralization-associated kinetic offsets are inconsistent with those seen at site F. Processes that produce DIC can indirectly cause KIEs associated with diffusive transport and kinetically limited chemical reactions. Because lighter isotopologues are diffused faster, the isotopic composition of residual carbonate can be affected as DIC is diffused away (Fig. 3, A and B, dotted line). Processes that consume CO_2_ [e.g., photosynthesis and certain forms of methane oxidation (*44*)] commonly result in an increase in CaCO_3_ saturation and perturb the chemical equilibrium state, where bicarbonate dehydration and dehydroxylation occur more rapidly than CO_2_ hydration and hydroxylation; KIEs in these reactions then produce an isotopically disequilibrated DIC pool, a state inherited by precipitating carbonate minerals (*26*, *27*, *45*).

Model runs shown in Fig. 3 (C to H) show the steady-state output of the COAD-MS model as it is run with different exchange rates at which seawater and methane-rich fluid are introduced to the box (λ values ranging between 100 s^−1^ and 10^−7^ s^−1^). Note that the steady-state output fully accounts for KIEs that can occur during mineral precipitation, (de)hydration and (de)hydroxylation, and diffusive transport of DIC (β value set to 0.08 as default value). These models simulate initially rapid exchange rates with insufficient time for any chemical reactions to occur, and thus, the isotopic state represents equilibrium. The lines in these figures show the steady-state output of these models. They radiate from an equilibrium point (λ = 100 s^−1^) and are run with progressively smaller λ values, indicated by labeled contours and the direction of the arrowhead. As λ is reduced, AeOM and SD-AOM are given sufficient time to occur, driving the δ^18^O-∆_47_–∆_48_ system toward disequilibrium values. Increasing the concentration of methane in the sediment at seeps results in larger degrees of disequilibrium (Fig. 3, C and D). As the model is run with even slower exchange rates, DIC has sufficient time to begin to isotopically re-equilibrate, and thus, steady-state mineralization occurs closer to ∆_47_/∆_48_ equilibrium for model runs with λ values less than ~10^−5^ s^−1^. Thus, in an unknown system, the ∆_47_/∆_48_ disequilibrium could only ever yield a minimum estimate for the rate of SD-AOM (Fig. 3D), as the initial departure for all models with increased methane flux falls along identical trajectories regardless of methane concentration. Because the COAD-MS model is zero-dimensional, it is not possible to convert this SD-AOM rate to methane flux, although such a model is feasible. The model assumes that the DIC released from AeOM and AOM reactions is at isotopic equilibrium with respect to the DIC species, water temperature, and δ^18^O value and that the subsequent processes driving isotopic disequilibrium are purely the response predicted by the COAD model (*26*) to these perturbations to DIC. It is possible that there are kinetic isotope biases associated with the production of DIC via methanotrophy, as “vital effects” are often invoked in microbial biogeochemical systems (*46*). However, the agreement between our models, which assume no such KIEs, and measured data shows that such effects are not necessary to describe this system.

Accounting for aerobic reactions complicates the interpretation of these systems. In the low-*P*o_2_ (partial pressure of oxygen) bottom waters of the South China Sea [[O_2_] = 0.1 mM (*47*)], AeOM would initially reduce pH from 7.7 to 7.6, after which SD-AOM increases pH and results in CaCO_3_ saturation (shown in the Supplementary Materials). Running the COAD-MS model with increased dissolved O_2_ concentration shows that AeOM adds a kinetic bias to the system (Fig. 3, E and F). The aggregate effect of AeOM appears to initially follow the equilibrium ∆_47_/∆_48_ line before SD-AOM becomes the dominant chemical reaction. This initial effect is nonexistent in models where the mass-transport coefficient (β) is zero, and it is greater as this coefficient is increased (Fig. 3, G and H), suggesting that mass-dependent fractionation of DIC during diffusion is driving this effect. The ~1.5°C discrepancy between the measured and extrapolated temperatures shown in Fig. 2C could be attributed to a small amount of AeOM alongside SD-AOM, although this discrepancy in temperatures is smaller than 1 SE of measurement uncertainty and thus cannot confidently be resolved with current precision. The empirical slope for ∆_47_/∆_48_ values in our measured authigenic carbonate from site F is notably steeper than the predicted slope for dehydration and dehydroxylation of bicarbonate (Fig. 3, A and B). We propose that this represents the contribution of mass-dependent diffusional fractionation of DIC escaping the system.

The isotopic compositions of authigenic minerals at Formosa Ridge reflect the contribution of many physical and chemical processes. Modeling reveals that the primary contributors to this disequilibrated state are a rate-limiting bicarbonate dehydration reaction, which is coupled with the SD-AOM reaction. If the rate of exchange with seawater (i.e., via diffusion or advective mixing) occurs at a similar rate to these reactions, and CaCO_3_ saturation is achieved, then this characteristic state of isotopic disequilibrium would be preserved in the precipitating minerals. Because diffusive flux is inversely proportional to distance, the depth below seafloor of the SMTZ can be assumed to exert some control over the rate of exchange between pore fluid and seawater, and thus, minerals forming at different depths will have different states of isotopic disequilibrium. It must be noted, however, that even longer residence times would be expected to eventually result in more equilibrated carbonates, as shown in Fig. 3 (C and D), as longer residence times would eventually give the DIC sufficient time to re-achieve δ^18^O-∆_47_–∆_48_ equilibrium. Factors governing chemistry and mineralogy in methane-derived carbonates vary with geological context, and thus, our interpretation of site F carbonates is not universally transferrable. Clumped isotope analyses in the Barents Sea and North Sea show similarly correlated disequilibrium ∆_47_ values and δ^18^O values in the same overall direction and magnitude as at site F, but with the critical distinction, that aragonite samples were the most disequilibrated (*29*). There are many factors that dictate which polymorph of CaCO_3_ precipitates from solution such as Mg^2+^/Ca^2+^, water/rock ratios, and temperature (*48*, *49*): Variability in the chemistry of methane-rich fluids could therefore promote the formation of aragonite or calcite in locally distinct patterns.

Recently published analyses of the dual-clumped isotopes of nonseep dolomite associated with microbial sulfate reduction showed that an offset in ∆_47_ and ∆_48_ values accompanied positive δ^34^S anomalies (*38*), and these data provide valuable context for our results, as these also formed in the presence of sulfate reduction. Unlike Formosa ridge, however, the sulfate reduction is driven by anaerobic oxidation of organic matter, rather than methane. Lu and Swart (*38*) find that this dolomite inherited the disequilibrated state of DIC. The Bahamian dolomite yielded more negative than expected ∆_48_ values, as opposed to our observation of more positive values. Previous publications have attributed negative ∆_48_ trends with CO_2_ introduction into solution with rate-limiting hydration and hydroxylation reactions before mineralization (*26*, *27*, *31*, *32*, *35*, *50*), as well as combinatorial effects due to isotopically dissimilar sources of oxygen to the CO_2_ molecule (*50*, *51*). Both of these mechanisms could be possible in the Bahamas, as organic material contains oxygen itself that may be dissimilar from that of formational water, and the anaerobic metabolism of organic material can release CO_2_. These results show that sulfate reduction can result in both positive and negative ∆_48_ anomalies, suggesting fundamentally different behavior when organic matter or methane are consumed.

In summary, this work represents the first attempt to use dual-clumped isotopes as a tool for studying methane seep systems. We have demonstrated that dual-clumped isotopes can readily be used to identify authigenic carbonate minerals with kinetic biases. We show that aragonite compositions were indistinguishable from isotopic equilibrium, and their δ^18^O and ∆_47_ values accurately record seep temperature and seawater composition. Calcite components were invariably kinetically biased and yielded higher temperature estimates. In the case of seep province Formosa Ridge, reaction and mineralization occur along a similar kinetic slope characteristic of bicarbonate dehydration/dehydroxylation, resulting in more negative ∆_47_ and more positive δ^18^O and ∆_48_ values, although with some contribution from mass-dependent diffusive fractionation. Numerical models simulating steady-state carbonate precipitation at methane seeps show trends similar to those observed for site F. Our results imply that paleotemperatures of relic seeps can be accurately reconstructed through linear regression of disequilibrium data and back-extrapolation to equilibrium, or by using disequilibrated ∆_47_/∆_48_ values as a criterion for ignoring kinetically biased samples. The correlation between δ^18^O_carbonate_ values and clumped isotope disequilibrium highlights that the observed variance in δ^18^O values at seeps can, in this case, be entirely attributed to KIEs and not due to variable contribution of clathrate-derived water, as has been previously speculated for this site and others. The utility of this tool is now demonstrated for a modern environment, and future work will seek to apply this method to other modern systems as well as ancient deposits.

## MATERIALS AND METHODS

### Formosa Ridge

Formosa Ridge (also referred to as site F) is an active methane seep located south of mainland China in the northern South China Sea. Remotely operated vehicle (ROV) surveys of the area reveal copious authigenic crusts associated with methanotrophy, as well as epibenthic communities of mollusks, echinoderms, sponges, annelids, fish, and arthropods (*52*). Carbon isotope analysis of these fauna reveals a food web dependent upon using seep methane, whose δ^13^C value is −70.3‰ (VPDB) (*53*). The microcrystalline carbonates used in this study were collected with Deep Submergence Vehicle (DSV) *Jiaolong* over several dives in 2013, with four additional samples of coarsely crystalline carbonate collected with the ROV *ROPOS* onboard the R/V *Tan Kah Kee* in 2018. Sample collection locations in site F are shown by dive number in Fig. 1 and listed in Table 1. Feng and Chen (*20*) report a temperature at this location of 3.5°C. The pH in this region of the South China Sea can vary between 7.4 and 7.7, with typical values around 7.7 (*54*).

### Clumped isotope analysis

Clumped isotope analyses are conducted following the methods outlined in detail by Bernecker *et al.* (*55*). Samples containing 10 ± 0.2 mg of CaCO_3_ are reacted with phosphoric acid at 90°C, and the resulting CO_2_ is subsequently purified of water and other contaminants using the Hofmann Auto Line (HAL). This CO_2_ is analyzed relative to a reference gas on a Thermo253+ dual-inlet mass spectrometer. Equilibrated gas standards were used to project the resulting ∆_47_ and ∆_48_ data into the carbon dioxide equilibrated scale (CDES-90). Carbonate standards are co-measured to ensure analytical stability. Oxygen isotopes are corrected for CaCO_3_-CO_2_ acid fractionation using the correction factors for calcite and aragonite (*56*).

As a test for sample contamination by other phases, two samples were cleaned using 3 wt % sodium hypochlorite and analyzed in addition to the nonbleached samples. The bleached samples were within 95% confidence of their “unbleached” ∆_47_ and ∆_48_ values, an observation consistent with the results of a hydrogen peroxide bleaching experiment recently presented by Lu and Swart (*38*). Additionally, we analyzed a mixture of Carrara marble and pyrite to test the effects of deliberate sulfide contamination, which likewise had no effect on ∆_47_ and ∆_48_ values. These data are shown in Table 1. From this, we concluded that isotopic trends observed at site F are not a result of contamination.

### Modeling

We have constructed a customized implementation of the COAD model (*26*), which includes dissolved sulfate, sulfide, molecular oxygen, alkalinity, calcium, and dissolved inorganic carbonate at a methane seep. This COAD-MS model differs from the original COAD model in that it handles the aforementioned species, allowing pH to vary during a model run in response to carbonate precipitation and methane chemistry, facilitated by tracking the total alkalinity of the system and using the CO2SYS (*57*) model to calculate instantaneous pH. Several other modifications to the COAD model are detailed in Supplementary Text.

The model itself is structured as a single, zero-dimensional box model where seawater and seep methane are introduced from unchanging boundary conditions. The stoichiometry of SD-AOM is modeled using the following reaction (*44*), which generates two moles of alkalinity and overall consumes one mole of methane and sulfate, yielding one additional mole of DIC

$$CH_{4}+SO_{4}^{2-}+CO_{2}\to 2\text{HC}O_{3}^{-}+H_{2}S$$ (1)

AeOM is modeled with the following equation (*44*), which yields no alkalinity change but produces one additional mole of DIC, and the resulting water is ignored in the model itself

$$CH_{4}+2O_{2}\to CO_{2}+2H_{2}O$$ (2)

When O_2_ is available, sulfide oxidizes to sulfate with the following reaction (*44*), which consumes two moles of alkalinity and oxygen, yielding one mole of sulfate

$$H_{2}S+2O_{2}+2\text{HC}O_{3}^{-}\to 2CO_{2}+SO_{4}^{2-}+2H_{2}O$$ (3)

The rate of exchange with seawater is governed by the exchange coefficient λ, which is a user-specified parameter in the model

$$d{\left[CO_{2}\right]}_{\text{box}}/dt=\lambda \left({\left[CO_{2}\right]}_{\text{seawater}}-{\left[CO_{2}\right]}_{\text{box}}\right)$$ (4)

Exchange with seawater is considered as a possible mechanism for mass-dependent isotopic fractionation of DIC, where the diffusive coefficient for a given isotopologue (mass *X*) is governed by its mass and the mass-fractionation coefficient β, shown here for CO_2_

$$\lambda_{X}=\lambda {\left(\frac{44}{X}\right)}^{\beta }$$ (5)

For DIC species, β can range between −0.04 and 0.17 for DIC species (*28*, *45*). By varying λ, different behaviors can be observed in the isotopic composition of the precipitating carbonate mineral. As λ is reduced, reactants are given more time to react, and thus, the system is considered to be more reaction-dominated. Figure 3 (C to H) shows the steady-state output for multiple model simulations where λ values range between 100 s^−1^ and ~10^−5^ s^−1^. λ values are displayed as labeled ticks or contours in these figures, with arrows pointing toward lower λ values. Changes in methane concentration (Fig. 3, C and D), dissolved O_2_ concentration (Fig. 3, E and F), and different mass-dependent fractionation coefficient (β) values (Fig. 3, G and H) are tested and shown to affect the isotopic composition of precipitating carbonate. Additional details on this model are provided in Supplementary Text.

### Statistical analysis

The displayed 95% confidence intervals for samples in Figs. 2 and 3 are calculated with fully propagated uncertainties from sample measurement and standardization (*58*), following the best practice implementation outlined by Bernecker *et al.* (*55*). Equilibrium versus nonequilibrium precipitation is defined by the criteria of the intersection of 95% confidence error bars with the dual-clumped isotope equilibrium line of Fiebig *et al.* (*34*). In Fig. 2, the linear regression is calculated using a least-squares regression, a process repeated in a Monte-Carlo simulation 10,000 times using re-generated datasets following the measured means and confidences of the measured carbonates. Each iteration yields an intercept with the equilibrium line, which is then used to calculate equilibrium δ^18^O_carbonate_ and δ^18^O_water_. The reported uncertainties for these parameters are calculated by the 2.5 and 97.5 percentiles of this final distribution, thus representing a cumulative 95% of the modeled values. A Matlab script is provided in the Supplementary Materials to show this process and produce Fig. 2 (https://zenodo.org/records/10954482).

## Supplementary Materials

### This PDF file includes:

Supplementary Text Figs. S1 to S9 Table S1 Legends for data S1 and S2 References
