# Supplementary material for: Resolving and correcting for kinetic biases on methane seep paleotemperature using carbonate ∆47/∆48 analysis
Source: Sci Adv. 2024 May 29;10(22):eadn0155. doi: 10.1126/sciadv.adn0155 (PMC11135390; doi:10.1126/sciadv.adn0155)
Supplement: Supplementary file 1 — Supplementary Text Figs. S1 to S9 Table S1 Legends for data S1 and S2 References [file sciadv.adn0155_sm.pdf]

Science Advances
AAAS

Supplementary Materials for

Resolving and correcting for kinetic biases on methane seep paleotemperature using carbonate Δ_{47}/Δ_{48} analysis

Philip Staudigel *et al.*

Corresponding author: Philip Staudigel, staudigel@em.uni-frankfurt.de

Sci. Adv. **10**, eadn0155 (2024)
DOI: 10.1126/sciadv.adn0155

The PDF file includes:

Supplementary Text
Figs. S1 to S9
Table S1
Legends for data S1 and S2
References

Other Supplementary Material for this manuscript includes the following:

Data S1 and S2

Supplementary Text

Setup and governing equations in the COAD-MS methane seep box model:

The COAD-MS model and the scripts used to generate Figures 2 and 3 can be accessed via the following *Zenodo* DOI: https://doi.org/10.5281/zenodo.11066480

The COAD-MS model simulates the chemical reactions governing dissolved inorganic carbon, oxygen, calcium, methane, sulfate, sulfide, total alkalinity and the carbonate minerals precipitating from the fluid. The default initial state of the model (except where otherwise specified) is an approximation of bottom water in the South China Sea, shown in Table S1.

Reaction equations and rates for methane – molecular oxygen – sulfate – sulfide

To model the sulfate-driven anaerobic oxidation of methane (SD-AOM) and aerobic oxidation of methane (AeOM) and the oxidation of sulfide, the following reactions (also shown in main text) are modeled in addition to those used in the original Watkins and Devriendt (2022) COAD model(*26*).

$$CH_4 + SO_4^{-2} + CO_2 \rightarrow 2HCO_3^- + H_2S \qquad \text{Equation 1}$$

$$CH_4 + 2O_2 \rightarrow CO_2 + 2H_2O \qquad \text{Equation 2}$$

$$H_2S + 2O_2 + 2HCO_3^- \rightarrow 2CO_2 + SO_4^{2-} + 2H_2O \qquad \text{Equation 3}$$

To summarize, for one mol of methane consumed, SD-AOM yields 1 mol of DIC and 2 mols of alkalinity and AeOM yields 1 mol of DIC an no alkalinity. The aerobic oxidation of sulfide consumes two mols of alkalinity, thus SD-AOM and subsequent oxidation of sulfide is essentially the same as simply aerobically oxidizing the methane from the perspective of carbonate saturation state and pH. The precipitation of $CaCO_3$ consumes two mols of alkalinity and 1 mol of DIC. The effects of these processes on solution pH and CaCO3 saturation state are summarized in Figure S1.

The above chemical reactions are assumed to release CO_2 and HCO_3^- that are at isotopic equilibrium with respect to carbon, oxygen and clumped isotopes, defined by the fractionation factors of Beck et al.(*59*), Mook(*60*), and Hill et al.(*61*). This assumption is taken in order to test if there is a fundamental necessity for microbial AOM-specific fractionation to produce the measured isotopic disequilibrium of precipitating carbonates. There are arguments that these fractionation effects may exist, as it is understood that microbial processes can produce DIC that is out of isotopic equilibrium(*46*, *62*). We argue that there is value in initially assuming that no such fractionation occurs and testing this system purely from the perspective of DIC reaction kinetics responding to a chemical perturbation. Our findings show that the observed variance and co-variance in the $\delta^{18}O$–Δ_{47}–Δ_{48} system can be readily described with this approach, demonstrating that there is no need for such additional reaction-specific fractionation factors.

A function, titled *Seep_ODE.m* has been constructed in Matlab, which computes the instantaneous rate of change of CO_2 (mass 44-48) Exchangeable Inorganic Carbon (EIC: HCO_3^-

$+CO_3^{2-}$ mass 60-64) as governed by the COAD model, as well as alkalinity, sulfate, sulfide and oxygen. The model uses the total concentration of dissolved inorganic carbon, and the alkalinity, to calculate the instantaneous pH using the CO2SYS model(*57*). Chemical transport with the boundary condition (seawater) is governed by the exchange rate, λ, which is constant for all chemical components and is governed by Fick's law for diffusive transport, where $C_{external}$ is either seawater or the methane supply.

$$Flux = (C_{external} - C_{box}) * \lambda \qquad \text{(Equation S1)}$$

Mass-dependent fractionation of isotopologues by this transport flux is allowed by varying λ following a mass-dependent relationship, shown by example for a mass-63 isotopologue of dissolved carbon.

$$\lambda_{63} = \lambda_{60}\left(\frac{60}{63}\right)^{\beta} \qquad \text{(Equation S2)}$$

In a purely kinetic system, average particle velocities (and thus their rate of dispersal) at a given temperature are dictated by the square root of their mass, thus β would be 0.5; due to interactions between particles, such as the hydration of ions by surrounding water molecules, this number is typically much less. According to molecular dynamics simulations systems, β for DIC constituents can be up to 0.17, (*28*, *45*). In the models shown in the main text, a range of values is shown in **Figure 3g-h** between 0 and 0.15, however the default value in these models is 0.08, which appears to yield agreeable results with measured values.

The functions provided as supplementary files by Watkins and Devriendt (2022) are modified slightly for this model. The first modification allows for a temperature-dependent saturation state of calcite, which is taken from *CO2SYS*(*57*) as the original script uses an invariant value calculated for 25°C. Further, slightly different polynomials approximating the results of Hill et al. (*61*) are used to define the equilibrium Δ_{63} and Δ_{64} of calcite, such that the model yields equilibrium calcite Δ_{47} and Δ_{48} values that are in exact agreement with the Δ_{47}/Δ_{48} calibration published recently by Fiebig et al. (2021)(*34*). Furthermore, the values for k_{+1}, a_{+1}, b_{+1}, c_{+1}, k_{+4}, a_{+4}, b_{+4}, c_{+4} are now taken from those used in the IsoDIC model(*27*), rather than the values chosen by Watkins and Devriendt(*26*). The saturation state of carbonate in the original COAD model was a fixed value from Jacobsen (1974) and was insensitive to temperature, we have replaced this with the temperature and salinity sensitive K_{sp} value of Mucci (*63*), which is the same equation used by the *CO2SYS.m* function. The polynomials describing equilibrium Δ_{63} and Δ_{64} in the *CaCO3_DIC.m* have been changed to match the Hill et al. (*61*) polynomials used by Fiebig et al. (*34*), thus the definition for isotopic equilibrium is precisely consistent between measured and modeled values.

The differential equation, *Seep_ODE.m*, is solved using Matlab's *ode23s* solver inside a function, *Seep_Function.m*, which defines the initial state of the model and boundary conditions. When the model is finished, the instantaneous $\delta^{13}C$, $\delta^{18}O$, Δ_{47}, Δ_{48} values of CO_2, EIC, $CaCO_3$, as well as the pH and dissolved sulfate, sulfide and methane abundance are returned by the function at specified timepoints. In the figures of the main text, each point on a given line represents one steady-state solution (the final state of a given model run), and thus each vector is a composite of multiple model runs with different values for λ.

Model results:
To demonstrate the overall behavior of the COAD-MS Model, the time-varying results are shown in Figure S2 for five model iterations with λ values of $10^{-1}s^{-1}$, $10^{-2.5}s^{-1}$, $10^{-4}s^{-1}$, $10^{-5}s^{-1}$, and $10^{-6}s^{-1}$.

As λ increases, more reaction chemistry is able to occur before products and reactants are exchanged with the boundary conditions, resulting in variable extents of kinetic offsets. Models show four distinct stages during the progression. 1) O_2 consumption, with corresponding increase in ∑DIC and decrease in pH. 2) SO_4^{2-} consumption, with corresponding increases in ∑DIC and pH, and offsets in isotope compositions. This occurs until 3) $CaCO_3$ precipitation occurs, which is associated with a more limited increase in pH, as well as consumption of ∑DIC, Ca^{2+}. 4) Attainment of a steady state, where no further change in chemistry occurs and all fluxes are balanced.

When the output steady state values for carbonate flux, pH and precipitating carbonate are plotted relative to λ, Figure S3 is generated. Figure S3 shows that there is an optimum rate of transport that allows carbonate precipitation to occur at an optimum rate, and that this optimum carbonate precipitation flux, also roughly coincides with the maximum kinetic offset in $\delta^{18}O$, Δ_{47} and Δ_{48}. Crossplotting these output isotopic values produces the diagrams shown in Figure 3c-h of the main text. By varying the methane flux, the exact magnitude of this offset can vary, as shown in Figure 3c-d of the main text.

Testing the effects of other parameters on the COAD-MS model:
The models discussed in the main text were implemented with model conditions that represented the present day at Site F, (i.e. Temperature = 3.5°C, salinity = 35 ppt, $[Ca^{2+}]$ = 10 mM, pH = 7.7). Testing the model's behavior at other temperatures, salinities, ion concentrations and pH values would give predictions for how these systems would behave in other locations and seawater chemistries. Here, we re-run the model used to generate Figures 3g and 3h of the main text, with different parameters.

Salinity affects a number of model behaviors, critically it is related to the saturation state of carbonate, as well as the rate of hydration and hydroxylation reactions. Increasing salinity results in lower equivalent saturation state of calcium carbonate, and thus higher concentrations of DIC are required to precipitate $CaCO_3$, resulting in diffusive fractionation manifesting itself more in these systems. Figure S4 tests the COAD-MS model using salinities of 20, 30 and 40 ppt. Lower salinities than 20 ppt result in super-saturation of $CaCO_3$ at the starting condition.

Temperature governs the equilibrium isotopic composition for $\delta^{18}O$, Δ_{47} and Δ_{48} of carbonate the model, however the rate constants for hydration/hydroxylation are both also related to temperature (*64*), thus warmer temperatures are associated with more rapid equilibration of the DIC pool, and thus with comparatively smaller kinetic offsets and different equilibrium states, this is shown in Figure S5, which tests the model at 0°C, 10°C and 20°C. The coefficients used for methanotrophy are not temperature-dependent, however if these are also increasing, then

kinetic offsets may still be expected at warmer temperatures due to faster rates of SD-AOM and AeOM.

Similarly, the rate of equilibration and the saturation state of carbonate are both affected by solution pH, which can also vary in the modern ocean between 7.6 and 8.2, but may have ranged between 6.5 to 9 throughout geologic history (*65*). We explore this in Figure S6 with simulations run with pH values of 7, 7.5 and 8. It is unclear how realistic independently varying pH is, as it is generally coupled in seawater with Ca and DIC concentrations via the solubility product of calcite or CO_2 removal and addition via photosynthesis or atmospheric exchange. Nevertheless, lower pH is associated with more rapid equilibration rates due to increased [CO_2] and [H^+] concentrations facilitating faster hydration reactions, and thus lower pH is associated with less kinetic offsets than higher pH. Solution pH has a secondary effect related to carbonate saturation state, in that DIC concentrations must be higher at lower pH in order to result in mineral formation, a consequence of this is an enhanced contribution of mass-dependent fractionation via diffusion in the lower pH model runs than higher pH runs.

The choice of kinetic rate parameters and the associated isotopic kinetic fractionation factors for hydration and hydroxylation, as well as the fractionation of oxygen isotopes between water and hydroxide has a demonstrable effect on model parameters. In Figure S7**,** we test the effects of these choices on model output. These parameters were taken from Guo (*27*) (Fig. S7a, and Figure 3 of main text), Watkins and Devriendt (*26*) (Fig. S7b), and Chen et al., (*66*) (Fig. S7c). All models subsequently use the same intrinsic clumped isotope fractionation factors, which are taken from Guo (*27*), as implemented by Watkins and Devriendt (*26*). Changing these parameters appears to result in different degrees of departure from Δ_{47}/Δ_{48} equilibrium, however with very similar overall behaviors. In the attached code (Data S2), it is possible to change between these parameters in the *loadEqs.m* script.

Testing for possible isobaric effects on Δ_{47}/Δ_{48} by sulfur and oxidizable contaminants:
Isobaric interference is a problem in natural samples with mixed compositions, as organic components and minerals can release gases during acidification that would interfere at the 44-49 amu range measured in CO_2 for clumped isotopes. Sulfide minerals are a common accessory mineral in methane seep deposits, as the sulfide derived from SD-AOM interacts with metal ions (e.g., Fe) to form minerals such as pyrite. The oxidation of such minerals during laboratory handling and analysis could potentially release sulfur oxide, whose most common isotopologue is $^{32}S^{16}O$, with a mass of 48. Thus, we deliberately sought out to test if this has had any measurable effect on the clumped isotope composition of our samples. This was done with two experiments, 1) deliberate contamination of a pure carbonate sample, and 2) bleaching of methane seep carbonate samples. This second experiment also serves as a test for other sources of isobaric interference as well.

A sample of Carrara marble was deliberately contaminated with 2 wt.% pyrite to test for these effects. Comparison between the contaminated and uncontaminated samples (shown in table 1 of the main text) showed no significant difference in isotopic composition, indicating that the presence of pyrite does not produce a measurable effect on the clumped isotope composition.

It has also been postulated that the presence of organic matter in carbonate samples may evoke a bias in clumped isotope values if not being removed prior to phosphoric acid digestion (e.g., Bergmann et al., 2018). To test if oxidative cleaning of samples has an effect, aliquots of Formosa Ridge carbonate were bleached for 24 hours in a 3wt.% sodium hypochlorite solution. Δ_{47} and Δ_{48} values of treated and untreated samples, however, were indistinguishable with 2SE, demonstrating that these samples are unlikely to be affected by isobaric contamination (Table 1).

Expressing isotopologue models' output as dδ/dt or dΔ/dt

The numerical models presented by Chen et al.(*66*), Uchikawa et al.(*67*), Watkins and Devriendt(*26*), Guo(*27*), and others calculate the instantaneous rate of change of the many isotopologues of dissolved inorganic carbon. Performing these calculations while tracking isotopologues as independent species makes calculation of isotopologue mixing and kinetic isotope effects much simpler. However, when interpreting these results, it is often helpful to re-calculate the output values in delta-notation. The math governing this has been presented before with the following equations (where the isotopologue weight of a carbonate ion is given in brackets), in this case omitting the contribution of ^{17}O isotopologues at each mass following the conventions of Chen *et al.*(*66*), Uchikawa *et al.*(*67*) and Watkins and Devriendt(*26*).

$$\delta^{13}C_{PDB} = \frac{[61]}{[60]} \times \frac{1000}{R_{PDB}} - 1000 \qquad \text{(Equation S3)}$$

$$\delta^{18}O_{PDB} = \frac{[62]}{[60]} \times \frac{1000}{3R_{PDB}} - 1000 \qquad \text{(Equation S4)}$$

$$\Delta_{63} = \frac{[63][60]}{[61][62]} \times 1000 - 1000 \qquad \text{(Equation S5)}$$

$$\Delta_{64} = \frac{[64][60]}{[62][62]} \times 3000 - 1000 \qquad \text{(Equation S6)}$$

If the rate of change for each isotopologue (written here as [X]') can be calculated using a model, for instance as shown for mass-dependent diffusive fractionation earlier in this supplementary file, then the rate of change of $\delta^{13}C$, $\delta^{18}O$, Δ_{63} and Δ_{64} (written here as $\delta^{13}C'$ $\delta^{18}O'$ Δ_{63}' and Δ_{64}') can be calculated using the Constant Rule, Constant Multiple Rule, Product Rule and Quotient Rule to the following derivatives.

$$\delta^{13}C' = \frac{[61]'[60]-[61][60]'}{[60]^2} \times \frac{1000}{R_{PDB}} \qquad \text{(Equation S7)}$$

$$\delta^{18}O' = \frac{[62]'[60]-[62][60]'}{[60]^2} \times \frac{1000}{3R_{PDB}} \qquad \text{(Equation S8)}$$

$$\Delta_{63}' = \frac{[62][61]([63]'[60]+[63][60]')-[63][60]([62]'[61]+[62][61]')}{([61][62])^2} \times 1000 \qquad \text{(Equation S9)}$$

$$\Delta_{64}' = \frac{[62]([64]'[60]+[64][60]')-2[62]'[64][60]}{[62]^3} \times 3000 \qquad \text{(Equation S10)}$$

Converting the Δ_{63} and Δ_{64} values from equations S5, S6 into the more conventionally displayed CO_2 Δ_{47} and Δ_{48} values for calcium carbonate is performed with the empirical linear transformations from Fiebig *et al.,* (*34*).

$$\Delta_{47} = 1.038 \times \Delta_{63} + 0.1856 \qquad \text{(Equation S11)}$$

$$\Delta_{48} = 1.028 \times \Delta_{64} + 0.1245 \qquad \text{(Equation S12)}$$

The derivatives of these terms, are similarly defined however with the constants omitted.

$$\Delta'_{47} = 1.038 \times \Delta'_{63} \qquad \text{(Equation S13)}$$

$$\Delta'_{48} = 1.028 \times \Delta'_{64} \qquad \text{(Equation S14)}$$

The relative rates of change of these parameters give the slopes of the lines for diffusive fractionation, CO_2 dehydration and CO_2 dehydroxylation shown in figure 3a and 3b in the main text.

Isotopologue end-member mixing: what (equilibrium) end-members could account for the observed variation in $\delta^{13}C$, $\delta^{18}O$, Δ_{47} and Δ_{48}?

Non-linear mixing effects in clumped isotopes are predictable and mathematically well-described within the published literature having been explicitly described for Δ_{47} by Defliese and Lohmann (*68*). Several studies have since presented theoretical and experimental tests of mixing for the dual-clumped isotope system (*33*, *38*, *69*). These non-linear mixing effects are a consequence of how the Δ_{47} and Δ_{48} parameters are calculated, as they are defined relative to the stochastic abundance of isotopes, which is the product of two values, represented using the reduced isotopologue approximation used by Watkins and Devriendt this is shown as $\Delta_{47} = 1000\ R_{47}/(R_{45}R_{46}) - 1000$. Because mixing lines between two end-members with different R_{45} and R_{46} values will have calculated stochastic R_{47} values that are parabolic in shape, whereas the mixing line of measured R_{47} values will be linear, there is an offset in these lines that appears to be a non-linear effect when only the Δ_{47} value is plotted, even though each individual isotopologue as behaves as a conservative "linear" mixing system. Whereas Δ_{47} mixing effects can be either positive or negative, Δ_{48} only ever exhibits positive mixing effects. It is possible to tune end-member $\delta^{13}C$ and $\delta^{18}O$ values to produce any measured slope of Δ_{47} and Δ_{48} values, such an exercise can also yield a minimum degree of internal heterogeneity necessary to produce a measured effect (*38*, *69*).

The variance in $\delta^{13}C$ and $\delta^{18}O$ values of carbonates at Site F have given previous workers reason to expect variable sources of carbon and water. Determining the possible contribution of end-member mixing at Site F provides a simple hypothetical question: can end-member mixing produce the observed disequilibrium effects in carbonate Δ_{47} and Δ_{48} values at Site F? In this exercise, it is necessary to assume that Δ_{47} and Δ_{48} values are at equilibrium, as not assuming this would necessitate the invocation of further kinetic isotope effects, which would negate the hypothesis. We further assume the equilibrium (clumped) temperatures of both endmembers are

identical, that is to say heterogeneity is driven by the bulk composition of the end-members, this is done to minimize the degrees of freedom in our model. Because the observed Δ_{47} values are more negative, the $\delta^{18}O$ and $\delta^{13}C$ values must be anti-correlated with one another (*68*). To yield the observed slope of Δ_{47} and Δ_{48} values, the difference in end-member $\delta^{18}O$ values must be roughly −0.2 to −0.6 the difference in end-member $\delta^{13}C$ values. From this, we modeled mixtures with $\Delta\delta^{13}C$ values of −25‰ from a starting $\delta^{13}C$ value of −45‰ and differences in $\delta^{18}O$ of −5‰ −10‰ and −5‰ from an equilibrium value of 3.5‰.

In Figure S8, we show end-member mixing models that would be sufficient to describe the measured seep composition. The $\delta^{13}C$ values of the modeled end-members are reasonable for methane-derived carbonate in this region, which has been measured at −70‰ (*53*). These mixing lines are plotted relative to the measured data in Figure S8. These models produced a sufficient mixing effect to describe the observed dataset, and necessitate an end-member water composition approximately 10‰ enriched relative to seawater. Experimental work at 4°C has shown that water in methane hydrate is enriched by 1.7‰–2.7‰ relative to surrounding water (*19*), thus methane hydrate-associated water is not a plausible source of such profoundly $\delta^{18}O$-enriched water. Clay mineral dehydration and mixing with meteoric water both constitute end-members with sufficiently dissimilar $\delta^{18}O$ to seawater to account for such mixing effects (*70*); we conclude that these mixing effects are a factor worth considering, particularly in systems where $\delta^{18}O$ can be extremely variable, such as with freshwater-seawater mixing zones.

Figure S9 explores mixing in freshwater-seawater mixing zones. We simulate a methane-derived carbon in a freshwater system mixing with seawater-derived DIC ($\Delta\delta^{13}C$ = −25‰VPDB; $\Delta\delta^{18}O$ = −5‰, −10‰, −15‰ VPDB). Results show that this system has equally extreme mixing effects, with a very positive deviation in Δ_{47} and Δ_{48}.

In the analysis of the resulting carbonate systems, these mixing effects could be resolved in several circumstances, A) if carbonate derived from both end-members is present in the sediment, which is then homogenized and analyzed. B) if DIC from both end-members is mixed and subsequently mineralized more rapidly than the time required for the isotopic re-equilibration of the DIC.

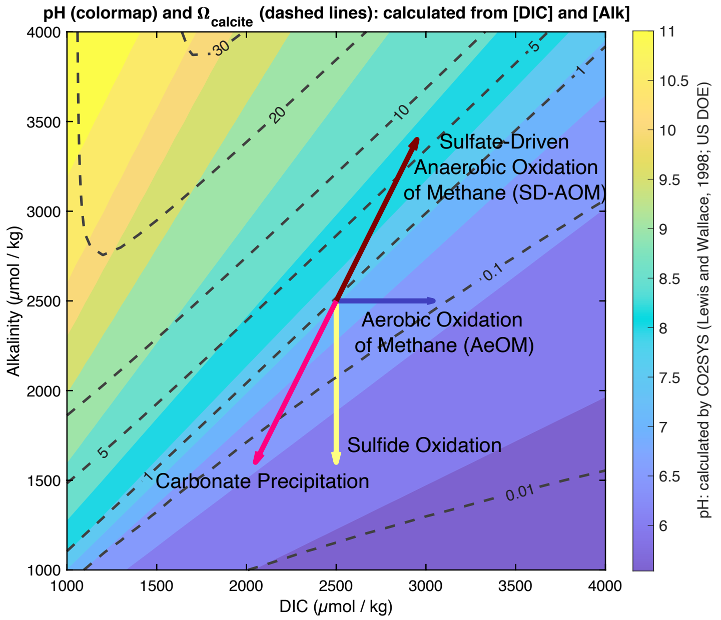

Fig. S1.

Alkalinity/DIC/pH/$\Omega_{calcite}$ plot with vectors showing the net effect of aerobic oxidation of methane (AeOM), anaerobic oxidation of methane (AOM), aerobic oxidation of sulfide, and carbonate precipitation. Color map shows pH of solution as calculated using CO2SYS(*57*). Dashed contours show saturation state of calcite, as calculated using CO2SYS(*57*), assuming unchanging seawater Ca^{2+} concentration of 10mM. All calculations use a salinity of 35ppt at 3.5°C at 1100 meters below sea surface.

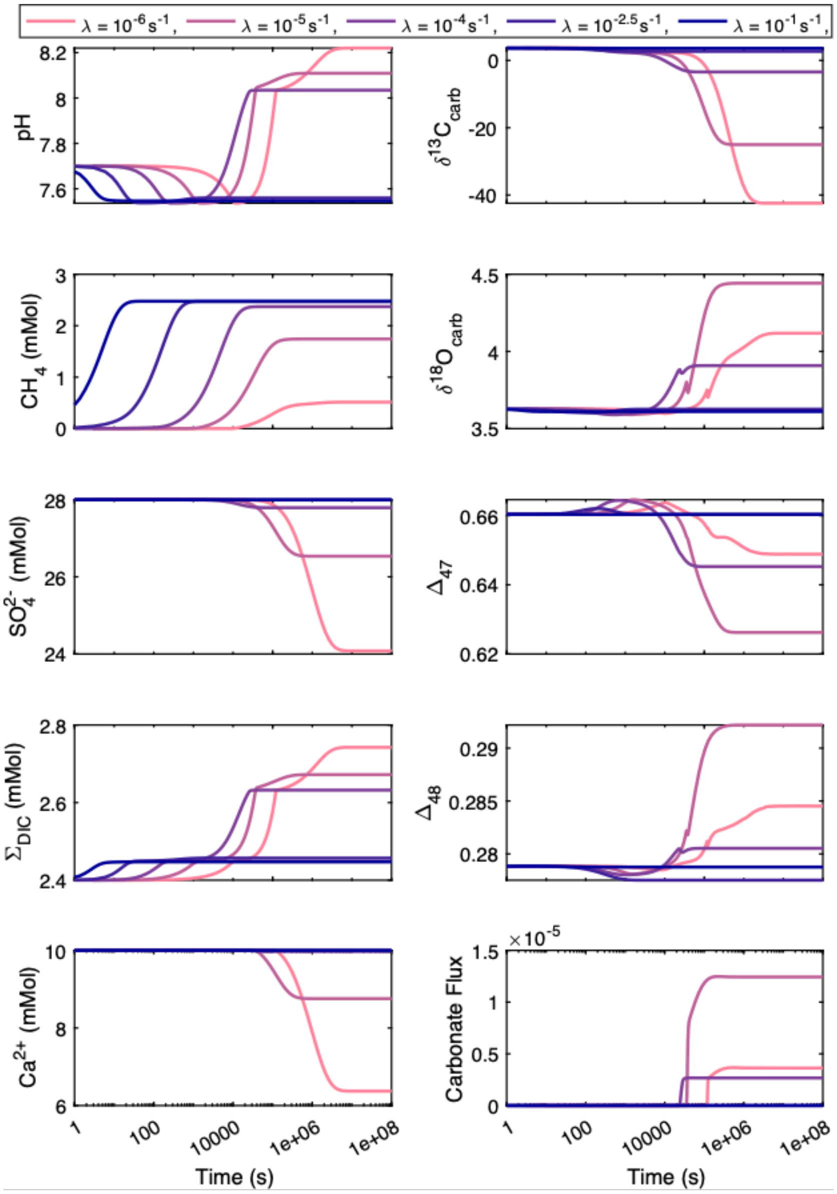

Fig. S2.

Water chemistry, and instantaneously precipitating carbonate isotopic composition for five model simulations with variable water exchange rates (lambda).

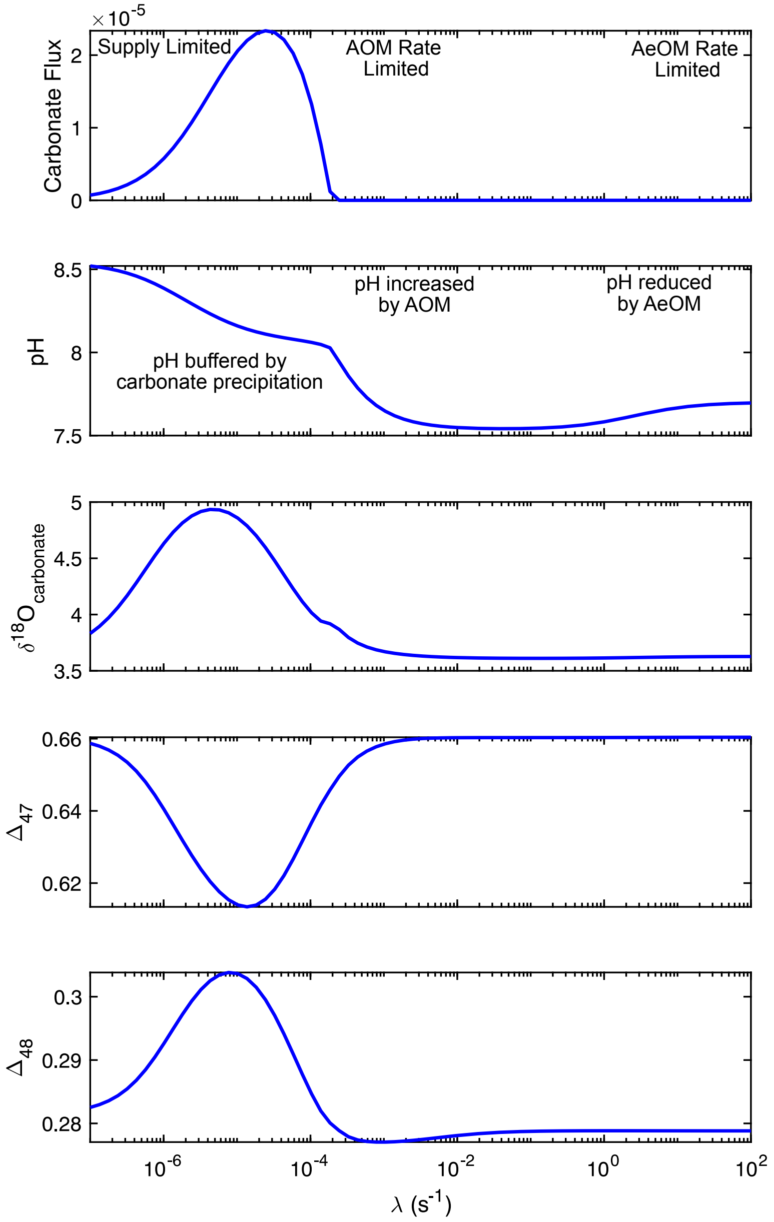

Fig. S3.
Water pH, and steady-state precipitating carbonate δ^{18}O, $_{\Delta 47}$ and Δ_{48} values relative to λ.

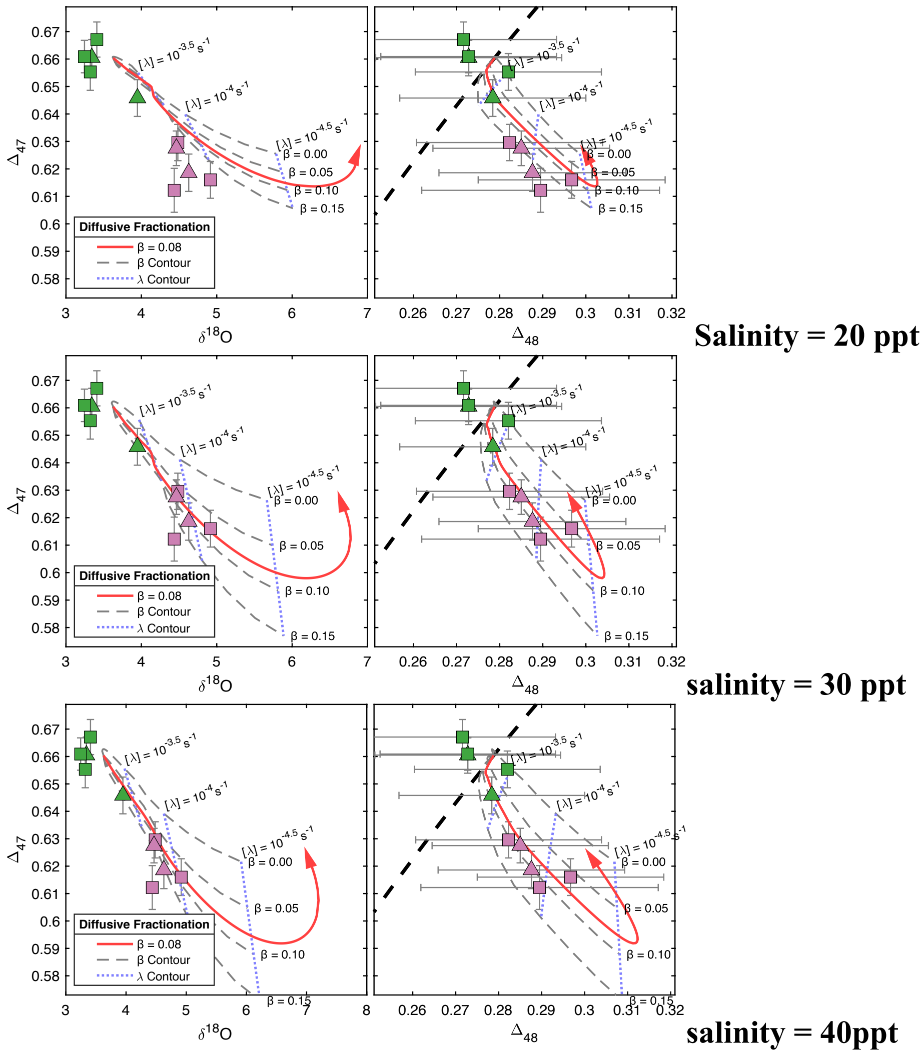

Fig. S4.
Model runs identical to Figure 3g,h of the main text, but with varying model salinity.

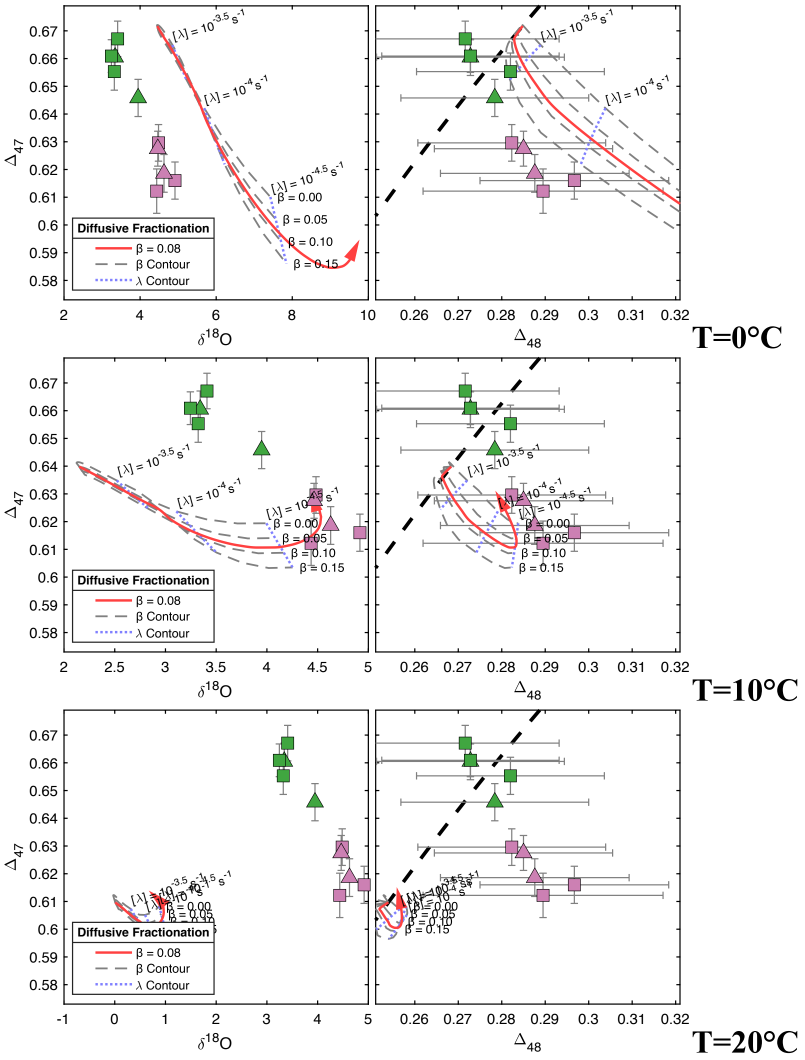

Fig. S5.

Model runs identical to Figure 3g,h of the main text, but with varying model temperature.

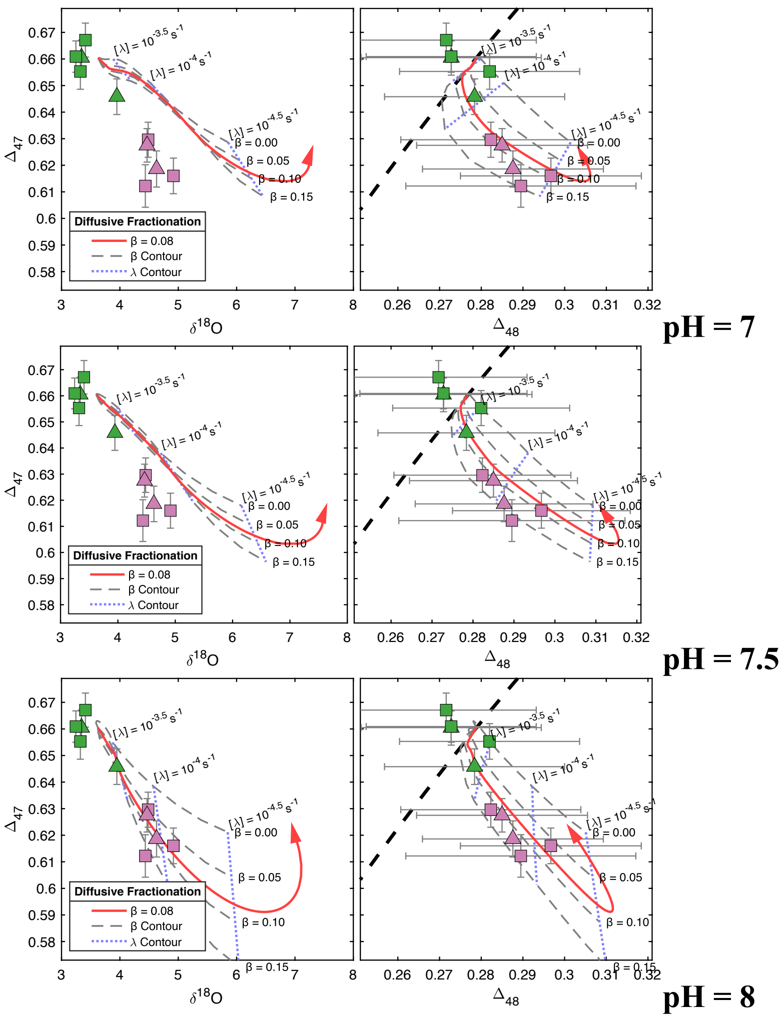

Fig. S6.
Model runs identical to Figure 3g,h of the main text, but with variable seawater boundary condition pH.

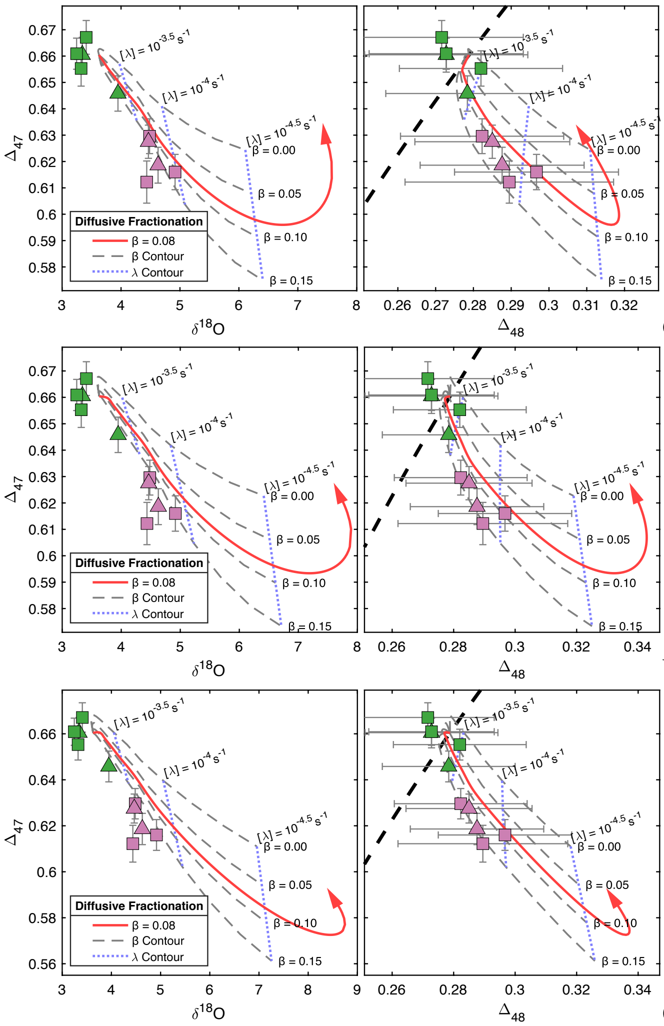

Guo (2021) parameters

Watkins and Devriendt (2021) parameters

Chen et al., (2018) parameters

Fig. S7.

Model runs identical to Figure 3g,h of the main text, but with different values for k_{+1}, k_{+4} (and associated kinetic isotope effects), as well as the $\alpha_{OH\text{-}H2O}$. Parameters taken from Guo (*27*), Watkins and Devriendt(*26*), Chen et al., (*66*), and the associated references therein.

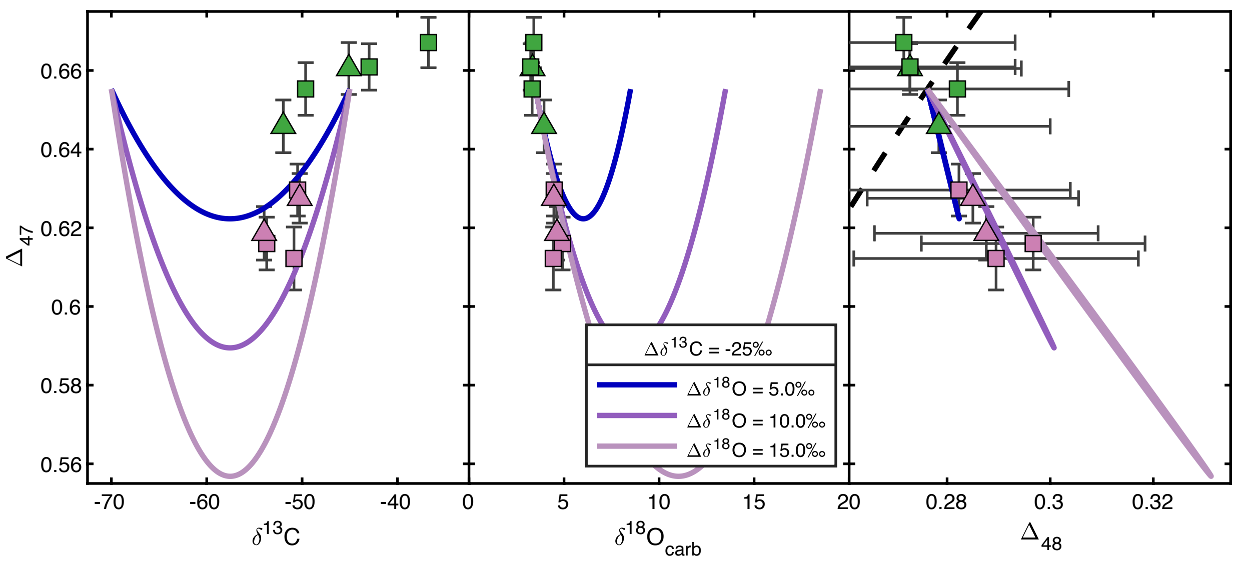

Fig. S8. Three mixing lines between the extrapolated equilibrium end-member, and hypothetical end-members with anti-correlated differences in $\delta^{13}C$ and $\delta^{18}O$, but identical Δ_{47} and Δ_{48} values.

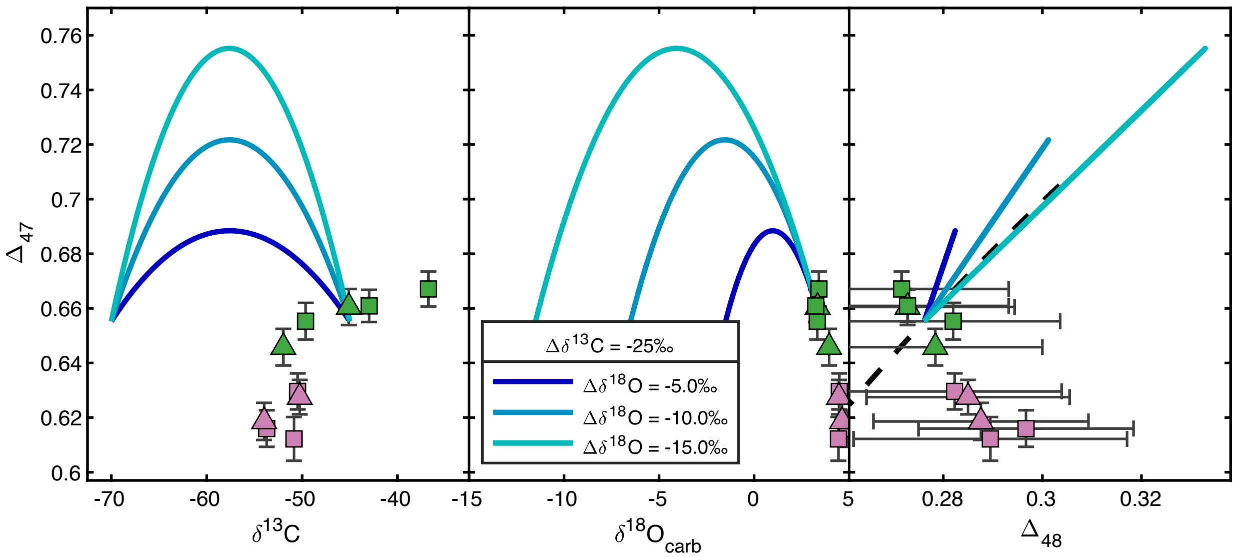

Fig. S9. Three mixing models between endmembers with correlated $\delta^{13}C$ and $\delta^{18}O$ values. Analogue for seawater and freshwater mixing zones with methane-derived carbon more abundant in the freshwater endmember.

Table S1.

Default composition of initial model state, and boundary condition seawater flux during model run.

**Property**	Value	Reference
Temperature	3.5 °C	Feng and Chen (2015)(*20*)
pH	7.7	Shao et al. (2016)(*54*)
Salinity	35 PSU	World Ocean Atlas (2018)(*47*)
$[O_2]$	0.1 mM	World Ocean Atlas (2018)(*47*)
$\sum_{DIC}$	2.4 mM	Cole et al. (2021)(*71*)
$[Ca^{2+}]$	10 mM	Pilson et al. (2012)(*72*)
$[SO_4^{2-}]$	28 mM	Halevy et al. (2012)(*73*)
$[H_2S]$	0	Pilson et al. (2012)(*72*)
Water $\delta^{18}O$	–0.3‰	Feng and Chen (2015)(*20*)
Seep $[CH_4]$	8 mM	Arbitrary user-specified variable
Methane $\delta^{13}C$	–70.3‰	Feng et al. (2015)(*53*)

Data S1. (separate file)

Excel table containing raw data for clumped isotope analyses used in this study, alongside anonymized unrelated data that was used for error minimization following Daëron (*58*) and Bernecker *et al.* (*55*).

Data S2. (separate file)

.zip file containing codes, data, and models used to generate Figure 2 and 3 of the main text. Also available via the following *Zenodo* DOI. https://doi.org/10.5281/zenodo.10954482

REFERENCES AND NOTES

1. K. A. Kvenvolden, T. D. Lorenson, The global occurrence of natural gas hydrate, in *Geophysical Monograph Series* (Blackwell Publishing Ltd., 2013), vol. **124**, pp. 3–18.

2. A. V. Milkov, Global estimates of hydrate-bound gas in marine sediments: How much is really out there? *Earth Sci. Rev.* **66**, 183–197 (2004).

3. B. A. Buffett, Clathrate hydrates. *Annu. Rev. Earth Planet. Sci*. **28**, 477–507 (2000).

4. E. Piñero, M. Marquardt, C. Hensen, M. Haeckel, K. Wallmann, Estimation of the global inventory of methane hydrates in marine sediments using transfer functions. *Biogeosciences* **10**, 959–975 (2013).

5. K. Wallmann, E. Pinero, E. Burwicz, M. Haeckel, C. Hensen, A. Dale, L. Ruepke, The global inventory of methane hydrate in marine sediments: A theoretical approach. *Energies (Basel)* **5**, 2449–2498 (2012).

6. K. A. Kvenvolden, Gas hydrates—Geological perspective and global change. *Rev. Geophys.* **31**, 173–187 (1993).

7. M. Maslin, M. Owen, S. Day, D. Long, Linking continental-slope failures and climate change: Testing the clathrate gun hypothesis. *Geology* **32**, 53–56 (2004).

8. K. Kretschmer, A. Biastoch, L. Rüpke, E. Burwicz, Modeling the fate of methane hydrates under global warming. *Global Biogeochem. Cycles* **29**, 610–625 (2015).

9. T. P. Fischer, A. Aiuppa, AGU centennial grand challenge: Volcanoes and deep carbon global CO_2 emissions from subaerial volcanism—Recent progress and future challenges. *Geochem. Geophys. Geosyst.* **21**, e2019GC008690 (2020).

10. P. Friedlingstein, M. O’sullivan, M. W. Jones, R. M. Andrew, L. Gregor, J. Hauck, C. Le Quéré, I. T. Luijkx, A. Olsen, G. P. Peters, W. Peters, J. Pongratz, C. Schwingshackl, S. Sitch, J. G. Canadell, P. Ciais, R. B. Jackson, S. R. Alin, R. Alkama, A. Arneth, V. K. Arora, N. R. Bates, M. Becker, N. Bellouin, H. C. Bittig, L. Bopp, F. Chevallier, L. P. Chini, M. Cronin, W. Evans, S. Falk, R. A. Feely, T. Gasser, M. Gehlen, T. Gkritzalis, L. Gloege, G. Grassi, N. Gruber, Ö. Gürses, I. Harris, M. Hefner, R. A. Houghton, G. C. Hurtt, Y. Iida, T. Ilyina, A. K. Jain, A. Jersild, K. Kadono, E. Kato, D. Kennedy, K.

Klein Goldewijk, J. Knauer, J. I. Korsbakken, P. Landschützer, N. Lefèvre, K. Lindsay, J. Liu, Z. Liu, G. Marland, N. Mayot, M. J. Mcgrath, N. Metzl, N. M. Monacci, D. R. Munro, S. I. Nakaoka, Y. Niwa, K. O'brien, T. Ono, P. I. Palmer, N. Pan, D. Pierrot, K. Pocock, B. Poulter, L. Resplandy, E. Robertson, C. Rödenbeck, C. Rodriguez, T. M. Rosan, J. Schwinger, R. Séférian, J. D. Shutler, I. Skjelvan, T. Steinhoff, Q. Sun, A. J. Sutton, C. Sweeney, S. Takao, T. Tanhua, P. P. Tans, X. Tian, H. Tian, B. Tilbrook, H. Tsujino, F. Tubiello, G. R. Van Der Werf, A. P. Walker, R. Wanninkhof, C. Whitehead, A. Willstrand Wranne, R. Wright, W. Yuan, C. Yue, X. Yue, S. Zaehle, J. Zeng, B. Zheng, Global carbon budget 2022. *Earth Syst. Sci. Data* **14**, 4811–4900 (2022).

11. M. De La Fuente, J. Vaunat, H. Marín-Moreno, Thermo-hydro-mechanical coupled modeling of methane hydrate-bearing sediments: Formulation and application. *Energies (Basel)* **12**, 2178 (2019).

12. G. R. Dickens, Down the rabbit hole: Toward appropriate discussion of methane release from gas hydrate systems during the Paleocene-Eocene thermal maximum and other past hyperthermal events. *Clim. Past* **7**, 831–846 (2011).

13. C. D. Ruppel, J. D. Kessler, The interaction of climate change and methane hydrates. *Rev. Geophys.* **55**, 126–168 (2017).

14. N. Li, X. Wang, J. Feng, F. Chen, Y. Zhou, M. Wang, T. Chen, G. Bayon, J. Peckmann, H. Cheng, Intermediate water warming caused methane hydrate instability in South China Sea during past interglacials. *Geol. Soc. Am. Bull.* **136**, 917–927 (2023).

15. S. Weldeab, R. R. Schneider, J. Yu, A. Kylander-Clark, Evidence for massive methane hydrate destabilization during the penultimate interglacial warming. *Proc. Natl. Acad. Sci. U.S.A.* **119**, e2201871119 (2022).

16. T. Himmler, D. Sahy, T. Martma, G. Bohrmann, A. Plaza-Faverola, S. Bünz, D. J. Condon, J. Knies, A. Lepland, A 160,000-year-old history of tectonically controlled methane seepage in the Arctic. *Sci. Adv.* **5**, eaaw1450 (2019).

17. K. Wallmann, M. Riedel, W. L. Hong, H. Patton, A. Hubbard, T. Pape, C. W. Hsu, C. Schmidt, J. E. Johnson, M. E. Torres, K. Andreassen, C. Berndt, G. Bohrmann, Gas hydrate dissociation off Svalbard induced by isostatic rebound rather than global warming. *Nat. Commun.* **9**, 83 (2018).

18. S. A. Akam, E. D. Swanner, H. Yao, W.-L. Hong, J. Peckmann, Methane-derived authigenic carbonates—A case for a globally relevant marine carbonate factory. *Earth Sci. Rev.* **243**, 104487 (2023).

19. D. W. Davidson, D. G. Leaist, R. Hesse, Oxygen-18 enrichment in the water of a clathrate hydrate. *Geochim. Cosmochim. Acta* **47**, 2293–2295 (1983).

20. D. Feng, D. Chen, Authigenic carbonates from an active cold seep of the northern South China Sea: New insights into fluid sources and past seepage activity. *Deep Sea Res. 2 Top. Stud. Oceanogr.* **122**, 74–83 (2015).

21. G. Bohrmann, J. Greinert, E. Suess, M. Torres, Authigenic carbonates from the Cascadia subduction zone and their relation to gas hydrate stability. *Geology* **26**, 647–650 (1998).

22. M. M. Savard, J. J. Jautzy, D. Lavoie, R. S. Dhillon, W. F. Defliese, Clumped and oxygen isotopes reveal differential disequilibrium in the formation of carbonates from marine methane seeps. *Geochim. Cosmochim. Acta* **298**, 43–54 (2021).

23. S. J. Loyd, J. Sample, R. E. Tripati, W. F. Defliese, K. Brooks, M. Hovland, M. Torres, J. Marlow, L. G. Hancock, R. Martin, T. Lyons, A. E. Tripati, Methane seep carbonates yield clumped isotope signatures out of equilibrium with formation temperatures. *Nat. Commun.* **7**, 1–12 (2016).

24. N. Thiagarajan, J. Adkins, J. Eiler, Carbonate clumped isotope thermometry of deep-sea corals and implications for vital effects. *Geochim. Cosmochim. Acta* **75**, 4416–4425 (2011).

25. R. E. Zeebe, D. Wolf-Gladrow, *CO_2 in Seawater: Equilibrium, Kinetics, Isotopes* (Gulf Professional Publishing, 2001).

26. J. M. Watkins, L. S. Devriendt, A combined model for kinetic clumped isotope effects in the $CaCO_3$-DIC-H_2O system. *Geochem. Geophys. Geosyst.* **23**, 2582–2594 (2022).

27. W. Guo, Kinetic clumped isotope fractionation in the DIC-H_2O-CO_2 system: Patterns, controls, and implications. *Geochim. Cosmochim. Acta* **268**, 230–257 (2020).

28. R. E. Zeebe, On the molecular diffusion coefficients of dissolved CO_2, HCO_3^-, and CO_3^{2-} and their dependence on isotopic mass. *Geochim. Cosmochim. Acta* **75**, 2483–2498 (2011).

29. N. Thiagarajan, A. Crémière, C. Blättler, A. Lepland, K. Kirsimäe, J. Higgins, H. Brunstad, J. Eiler, Stable and clumped isotope characterization of authigenic carbonates in methane cold seep environments. *Geochim. Cosmochim. Acta* **279**, 204–219 (2020).

30. J. Fiebig, D. Bajnai, N. Löffler, K. Methner, E. Krsnik, A. Mulch, S. Hofmann, Combined high-precision Δ_{48} and Δ_{47} analysis of carbonates. *Chem. Geol.* **522**, 186–191 (2019).

31. D. Bajnai, W. Guo, C. Spötl, T. B. Coplen, K. Methner, N. Löffler, E. Krsnik, E. Gischler, M. Hansen, D. Henkel, G. D. Price, J. Raddatz, D. Scholz, J. Fiebig, Dual clumped isotope thermometry resolves kinetic biases in carbonate formation temperatures. *Nat. Commun.* **11**, 1–9 (2020).

32. A. J. Davies, W. Guo, M. Bernecker, M. Tagliavento, J. Raddatz, E. Gischler, S. Flögel, J. Fiebig, Dual clumped isotope thermometry of coral carbonate. *Geochim. Cosmochim. Acta* **338**, 66–78 (2022).

33. P. Staudigel, A. J. Davies, M. Bernecker, M. Tagliavento, H. J. L. van der Lubbe, C. Nooitgedacht, N. Looser, S. M. Bernasconi, H. Vonhof, J. Fiebig, Fingerprinting kinetic isotope effects and diagenetic exchange reactions using fluid inclusion and dual-clumped isotope analysis. *Geochem. Geophys. Geosyst.* **24**, e2022GC010766 (2023).

34. J. Fiebig, M. Daëron, M. Bernecker, W. Guo, G. Schneider, R. Boch, S. M. Bernasconi, J. Jautzy, M. Dietzel, Calibration of the dual clumped isotope thermometer for carbonates. *Geochim. Cosmochim. Acta* **312**, 235–256 (2021).

35. A. J. Davies, U. Brand, M. Tagliavento, M. A. Bitner, D. Bajnai, P. Staudigel, M. Bernecker, J. Fiebig, Isotopic disequilibrium in brachiopods disentangled with dual clumped isotope thermometry. *Geochim. Cosmochim. Acta* **359**, 135–147 (2023).

36. P. K. Swart, C. Lu, E. W. Moore, M. E. Smith, S. T. Murray, P. T. Staudigel, A calibration equation between Δ_{48} values of carbonate and temperature. *Rapid Commun. Mass Spectrom.* **35**, e9147 (2021).

37. P. Staudigel, C. Pederson, J. van der Lubbe, M. Bernecker, M. Tagliavento, A. Davies, A. Immenhauser, J. Fiebig, An isotopologue-enabled model (Δ_{47}, Δ_{48}) for describing thermal fluid-carbonate interaction in open and closed diagenetic systems. *Geochem. Geophys. Geosyst.* **24**, e2023GC011117 (2023).

38. C. Lu, P. K. Swart, The application of dual clumped isotope thermometer (Δ_{47} and Δ_{48}) to the understanding of dolomite formation. *Geology* **52**, 56–60 (2024).

39. M. Tagliavento, A. J. Davies, M. Bernecker, P. T. Staudigel, R. R. Dawson, M. Dietzel, K. Götschl, W. Guo, A. S. Schulp, F. Therrien, Evidence for heterothermic endothermy and reptile-like eggshell mineralization in Troodon, a non-avian maniraptoran theropod. *Proc. Natl. Acad. Sci. U.S.A.* **120**, e2213987120 (2023).

40. S. T. Kim, J. R. O’Neil, C. Hillaire-Marcel, A. Mucci, Oxygen isotope fractionation between synthetic aragonite and water: Influence of temperature and Mg^{2+} concentration. *Geochim. Cosmochim. Acta* **71**, 4704–4715 (2007).

41. T. B. Coplen, Calibration of the calcite–water oxygen-isotope geothermometer at Devils Hole, Nevada, a natural laboratory. *Geochim. Cosmochim. Acta* **71**, 3948–3957 (2007).

42. D. Feng, Y. Peng, H. Bao, J. Peckmann, H. H. Roberts, D. Chen, A carbonate-based proxy for sulfate-driven anaerobic oxidation of methane. *Geology* **44**, 999–1002 (2016).

43. A. Haas, J. Peckmann, M. Elvert, H. Sahling, G. Bohrmann, Patterns of carbonate authigenesis at the Kouilou pockmarks on the Congo deep-sea fan. *Mar. Geol.* **268**, 129–136 (2010).

44. L. M. Wehrmann, S. Arndt, C. März, T. G. Ferdelman, B. Brunner, The evolution of early diagenetic signals in Bering Sea subseafloor sediments in response to varying organic carbon deposition over the last 4.3Ma. *Geochim. Cosmochim. Acta* **109**, 175–196 (2013).

45. W. Guo, C. Zhou, Patterns and controls of disequilibrium isotope effects in speleothems: Insights from an isotope-enabled diffusion-reaction model and implications for quantitative thermometry. *Geochim. Cosmochim. Acta* **267**, 196–226 (2019).

46. C. Thaler, A. Katz, M. Bonifacie, B. Menez, M. Ader, Oxygen isotope composition of waters recorded in carbonates in strong clumped and oxygen isotopic disequilibrium. *Biogeosciences* **17**, 1731–1744 (2020).

47. S. Spring, NOAA Atlas NESDIS 83 WORLD OCEAN ATLAS 2018 Volume 3: Dissolved oxygen, apparent oxygen utilization, and dissolved oxygen saturation (2019); https://www.ncei.noaa.gov/data/oceans/woa/WOA18/DATA/.

48. U. Balthasar, M. Cusack, Aragonite-calcite seas—Quantifying the gray area. *Geology* **43**, 99–102 (2015).

49. M. S. Hashim, S. E. Kaczmarek, The transformation of aragonite to calcite in the presence of magnesium: Implications for marine diagenesis. *Earth Planet. Sci. Lett.* **574**, 117166 (2021).

50. Z. A. Parvez, J. K. Lucarelli, I. W. Matamoros, J. Rubi, K. Miguel, B. Elliott, R. Flores, R. N. Ulrich, R. A. Eagle, J. M. Watkins, J. N. Christensen, A. Tripati, Dual carbonate clumped isotopes (Δ_{47}-Δ_{48}) constrains kinetic effects and timescales in peridotite-associated springs at the Cedars, Northern California, *Geochim. Cosmochim. Acta* **358**, 77–92 (2023).

51. L. Y. Yeung, Combinatorial effects on clumped isotopes and their significance in biogeochemistry. *Geochim. Cosmochim. Acta* **172**, 22–38 (2016).

52. Y. Zhao, T. Xu, Y. S. Law, D. Feng, N. Li, R. Xin, H. Wang, F. Ji, H. Zhou, J. W. Qiu, Ecological characterization of cold-seep epifauna in the South China Sea. *Deep Sea Res. 1 Oceanogr. Res. Pap.* **163**, 103361 (2020).

53. D. Feng, M. Cheng, S. Kiel, J. W. Qiu, Q. Yang, H. Zhou, Y. Peng, D. Chen, Using Bathymodiolus tissue stable carbon, nitrogen and sulfur isotopes to infer biogeochemical process at a cold seep in the South China Sea. *Deep Sea Res. 1 Oceanogr. Res. Pap.* **104**, 52–59 (2015).

54. C. Shao, Y. Sui, D. Tang, L. Legendre, Spatial variability of surface-sediment porewater pH and related water-column characteristics in deep waters of the northern South China Sea. *Prog. Oceanogr.* **149**, 134–144 (2016).

55. M. Bernecker, S. Hofmann, P. T. Staudigel, A. J. Davies, M. Tagliavento, N. Meijer, A. Ballian, J. Fiebig, A robust methodology for triple (Δ_{47}, Δ_{48}, Δ_{49}) clumped isotope analysis of carbonates. *Chem. Geol.* **642**, 121803 (2023).

56. S. T. Kim, A. Mucci, B. E. Taylor, Phosphoric acid fractionation factors for calcite and aragonite between 25 and 75 °C: Revisited. *Chem. Geol.* **246**, 135–146 (2007).

57. E. R. Lewis, D. W. R. Wallace, *Program Developed for CO_2 System Calculations* (Environmental System Science Data Infrastructure for a Virtual Ecosystem, 1998).

58. M. Daëron, Full propagation of analytical uncertainties in Δ_{47} measurements. *Geochem. Geophys. Geosyst.* **22**, e2020GC009592 (2021).

59. W. C. Beck, E. L. Grossman, J. W. Morse, Experimental studies of oxygen isotope fractionation in the carbonic acid system at 15°, 25°, and 40°C. *Geochim. Cosmochim. Acta* **69**, 3493–3503 (2005).

60. W. G. Mook, ^{13}C in atmospheric CO_2. *Neth. J. Sea Res.* **20**, 211–223 (1986).

61. P. S. Hill, A. K. Tripati, E. A. Schauble, Theoretical constraints on the effects of pH, salinity, and temperature on clumped isotope signatures of dissolved inorganic carbon species and precipitating carbonate minerals. *Geochim. Cosmochim. Acta* **125**, 610–652 (2014).

62. C. Thaler, C. Millo, M. Ader, C. Chaduteau, F. Guyot, B. Ménez, Disequilibrium $\delta^{18}O$ values in microbial carbonates as a tracer of metabolic production of dissolved inorganic carbon. *Geochim. Cosmochim. Acta* **199**, 112–129 (2017).

63. A. Mucci, The solubility of calcite and aragonite in seawater at various salinities, temperatures, and one atmosphere total pressure. *Am. J. Sci.* **283**, 780–799 (1983).

64. K. G. Schulz, U. Riebesell, B. Rost, S. Thoms, R. E. Zeebe, Determination of the rate constants for the carbon dioxide to bicarbonate inter-conversion in pH-buffered seawater systems. *Mar. Chem.* **100**, 53–65 (2006).

65. I. Halevy, A. Bachan, The geologic history of seawater pH. *Science* **355**, 1069–1071 (2017).

66. S. Chen, A. C. Gagnon, J. F. Adkins, Carbonic anhydrase, coral calcification and a new model of stable isotope vital effects. *Geochim. Cosmochim. Acta* **236**, 179–197 (2018).

67. J. Uchikawa, S. Chen, J. M. Eiler, J. F. Adkins, R. E. Zeebe, Trajectory and timescale of oxygen and clumped isotope equilibration in the dissolved carbonate system under normal and enzymatically-catalyzed conditions at 25 °C. *Geochim. Cosmochim. Acta* **314**, 313–333 (2021).

68. W. F. Defliese, K. C. Lohmann, Non-linear mixing effects on mass-47 CO_2 clumped isotope thermometry: Patterns and implications. *Rapid Commun. Mass Spectrom.* **29**, 901–909 (2015).

69. J. H. White, W. F. Defliese, $\delta^{13}C$ and $\delta^{18}O$ heterogeneities in carbonates: Nonlinear mixing in the application of dual-carbonate-clumped isotope thermometer. *Rapid Commun. Mass Spectrom.* **37**, e9627 (2023).

70. A. Micallef, M. Person, C. Berndt, C. Bertoni, D. Cohen, B. Dugan, R. Evans, A. Haroon, C. Hensen, M. Jegen, Offshore freshened groundwater in continental margins. *Rev. Geophys.* **59**, e2020RG000706 (2021).

71. J. J. Cole, O. Hararuk, C. T. Solomon, The carbon cycle: With a brief introduction to global biogeochemistry, in *Fundamentals of Ecosystem Science, Second Edition* (Elsevier, 2021), pp. 131–160.

72. M. E. Q. Pilson, *An Introduction to the Chemistry of the Sea* (Cambridge Univ. Press, 2012).

73. I. Halevy, S. E. Peters, W. W. Fischer, Sulfate burial constraints on the Phanerozoic sulfur cycle. *Science* **337**, 331–334 (2012).
